# Supplementary figures and images for: Rottlerin inhibits macropinocytosis of Porcine Reproductive and Respiratory Syndrome Virus through the PKCδ-Cofilin signaling pathway
Source: PLoS One. 2025 May 20;20(5):e0324500. doi: 10.1371/journal.pone.0324500 (PMC12091787; doi:10.1371/journal.pone.0324500)

Fig 1B

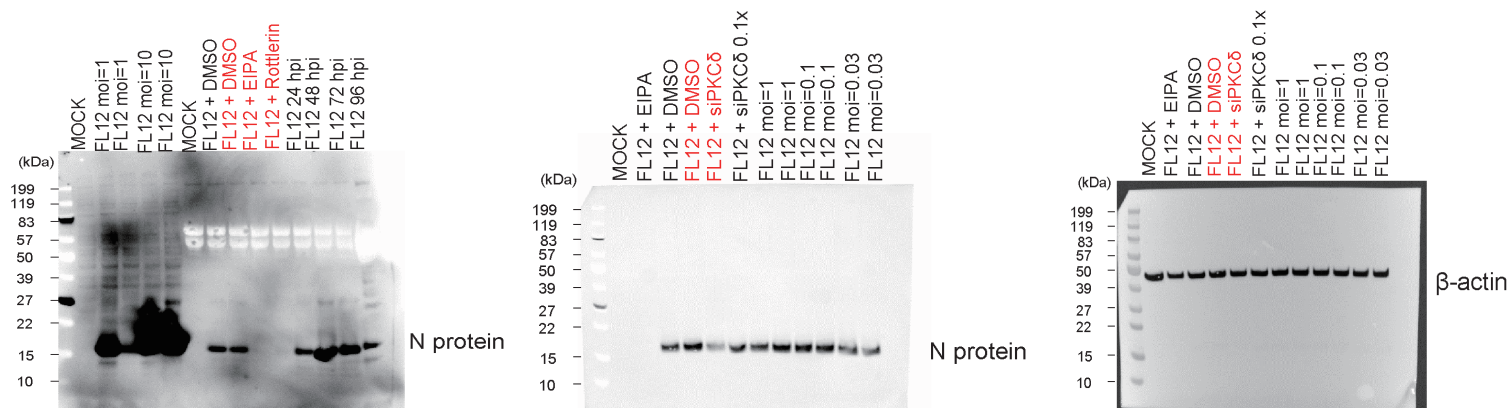

Fig 3A

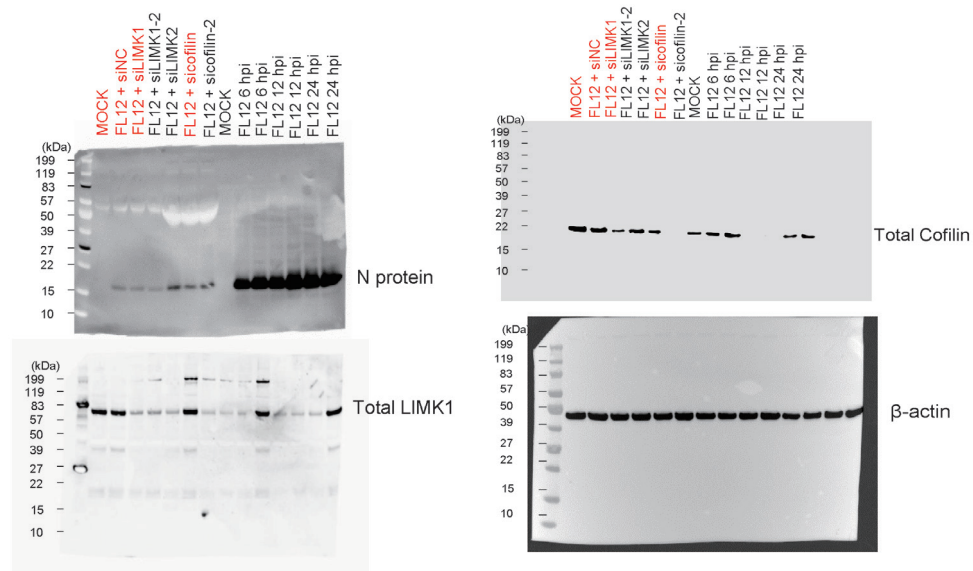

Fig 3C

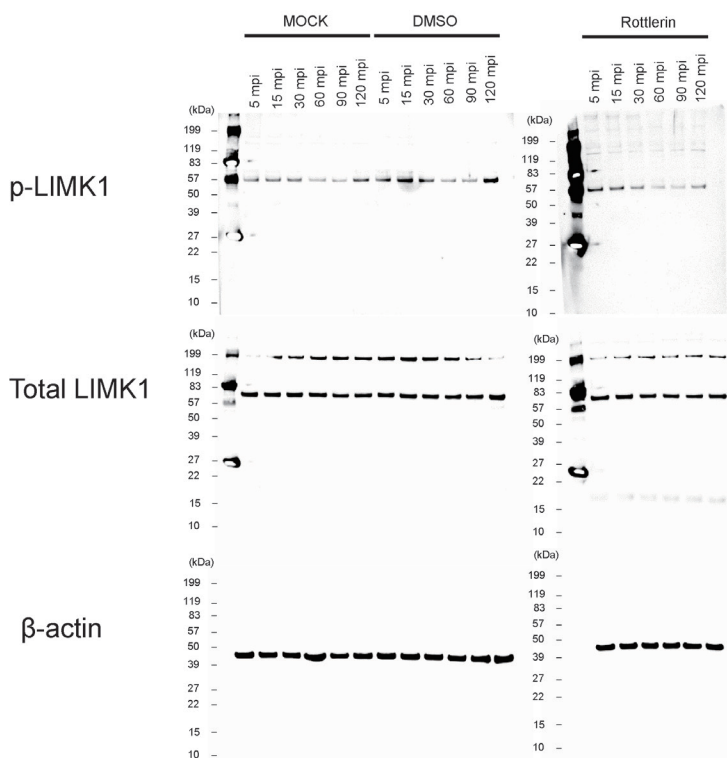

Fig 3E

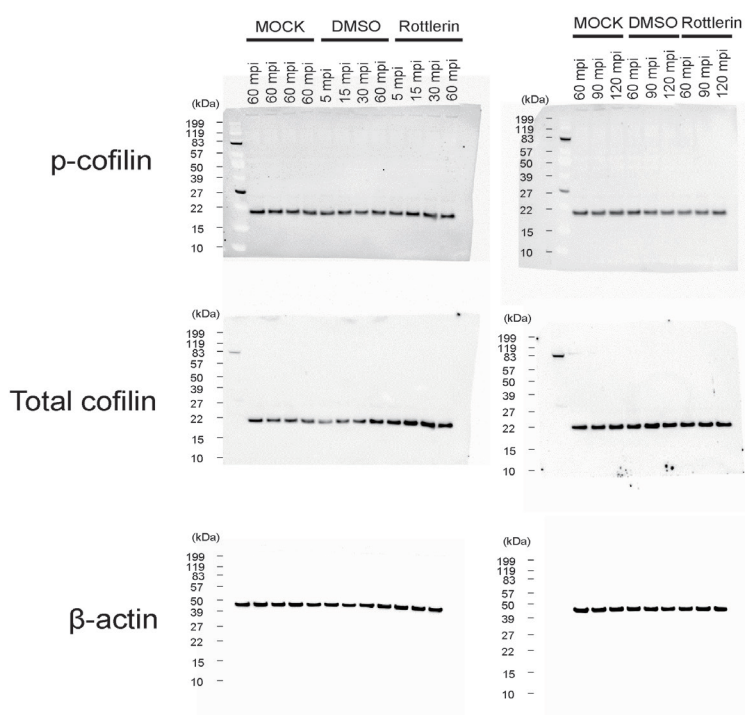

Supplement: S1 File — (PDF) [file pone.0324500.s001.pdf]
