# Supplementary material for: Rottlerin inhibits macropinocytosis of Porcine Reproductive and Respiratory Syndrome Virus through the PKCδ-Cofilin signaling pathway
Source: PLoS One. 2025 May 20;20(5):e0324500. doi: 10.1371/journal.pone.0324500 (PMC12091787; doi:10.1371/journal.pone.0324500)
Supplement: Attachment — Submitted filename: Response to Reviewers(Editor).doc [file pone.0324500.s005.doc]

PONE-D-24-40479

Rottlerin inhibits macropinocytosis of Porcine Reproductive and Respiratory Syndrome Virus through the PKCδ-Cofilin signaling pathway

PLOS ONE

Dear Dr. Gianmarco Ferrara,

Thank you for giving us the opportunity to submit a revised draft of the manuscript “Rottlerin inhibits macropinocytosis of Porcine Reproductive and Respiratory Syndrome Virus through the PKCδ-Cofilin signaling pathway” for publication in PLOS ONE. We appreciate the time and effort that you dedicated to providing feedback on our manuscript based on journal requirements. We have incorporated all of your suggestions. Those changes are highlighted within the manuscript. Please see below, in red, for a point-by point response to the editor’s comments.

1. Please ensure that your manuscript meets PLOS ONE's style requirements, including those for file naming. The PLOS ONE style templates can be found at

<https://journals.plos.org/plosone/s/file?id=wjVg/PLOSOne_formatting_sample_main_body.pdf> and

[https://journals.plos.org/plosone/s/file?id=ba62/PLOSOne_formatting_sample_title_authors_affiliations.pdf](https://nam12.safelinks.protection.outlook.com/?url=https%3A%2F%2Fc05y1x9s.r.us-east-2.awstrack.me%2FL0%2Fhttps%3A%252F%252Fjournals.plos.org%252Fplosone%252Fs%252Ffile%253Fid%3Dba62%252FPLOSOne_formatting_sample_title_authors_affiliations.pdf%2F1%2F010f01922c9ed281-4e21a2fa-e487-4379-82ed-176097abd6e1-000000%2F_voNHBtkhz2RF_ZxJbii__nerL4%3D178&data=05|02|changin.oh@yale.edu|09684ef41d5e4deb562008dcdde4f431|dd8cbebb21394df8b4114e3e87abeb5c|0|0|638629224138494093|Unknown|TWFpbGZsb3d8eyJWIjoiMC4wLjAwMDAiLCJQIjoiV2luMzIiLCJBTiI6Ik1haWwiLCJXVCI6Mn0%3D|0|||&sdata=U9Ahd2EF1SVJgxrTS6Hv%2FT0Z93PlkLWQrfezbbXgtE8%3D&reserved=0).

Author response: Thank you for pointing this out. I corrected them and highlighted.

2. Thank you for stating in your Funding Statement:

“This research was supported by the Korea Institute of Planning and Evaluation for Technology in Food, Agriculture, and Forestry (IPET) through the Agriculture, Food, and Rural Affairs Convergence Technologies Program for Educating the Creative Global Leader Program, funded by the Ministry of Agriculture, Food, and Rural Affairs (MAFRA) (320005-04).”

Please provide an amended statement that declares *all* the funding or sources of support (whether external or internal to your organization) received during this study, as detailed online in our guide for authors at <http://journals.plos.org/plosone/s/submit-now>.  Please also include the statement “There was no additional external funding received for this study.” in your updated Funding Statement.

Please include your amended Funding Statement within your cover letter. We will change the online submission form on your behalf.

Author response: I revised the funding statement as follows:

“This research was supported by the Korea Institute of Planning and Evaluation for Technology in Food, Agriculture, and Forestry (IPET) through the Agriculture, Food, and Rural Affairs Convergence Technologies Program for Educating the Creative Global Leader Program, funded by the Ministry of Agriculture, Food, and Rural Affairs (MAFRA) (320005-04).” There was no additional external funding received for this study.

3. Thank you for stating the following in the Acknowledgments Section of your manuscript:

“This research was supported by the Korea Institute of Planning and Evaluation for Technology in Food, Agriculture, and Forestry (IPET) through the Agriculture, Food, and Rural Affairs Convergence Technologies Program for Educating the Creative Global Leader Program, funded by the Ministry of Agriculture, Food, and Rural Affairs (MAFRA) (320005-04). We are deeply grateful to the KU Research Center for Zoonosis for providing advice on the design and progress of this study.”

We note that you have provided additional information within the Acknowledgements Section that is not currently declared in your Funding Statement. Please note that funding information should not appear in the Acknowledgments section or other areas of your manuscript. We will only publish funding information present in the Funding Statement section of the online submission form.

Please remove any funding-related text from the manuscript and let us know how you would like to update your Funding Statement. Currently, your Funding Statement reads as follows:

“This research was supported by the Korea Institute of Planning and Evaluation for Technology in Food, Agriculture, and Forestry (IPET) through the Agriculture, Food, and Rural Affairs Convergence Technologies Program for Educating the Creative Global Leader Program, funded by the Ministry of Agriculture, Food, and Rural Affairs (MAFRA) (320005-04).”

Please include your amended statements within your cover letter; we will change the online submission form on your behalf.

Author response: Thank you for pointing this out. I revised the Acknowledgements as follows. I revised the manuscript as well.:

We are deeply grateful to the KU Research Center for Zoonosis for providing advice on the design and progress of this study.

4. We note that your Data Availability Statement is currently as follows: [All relevant data are within the manuscript and its Supporting Information files.]

Please confirm at this time whether or not your submission contains all raw data required to replicate the results of your study. Authors must share the “minimal data set” for their submission. PLOS defines the minimal data set to consist of the data required to replicate all study findings reported in the article, as well as related metadata and methods (https://journals.plos.org/plosone/s/data-availability#loc-minimal-data-set-definition).

For example, authors should submit the following data:

- The values behind the means, standard deviations and other measures reported;

- The values used to build graphs;

- The points extracted from images for analysis.

Authors do not need to submit their entire data set if only a portion of the data was used in the reported study.

If your submission does not contain these data, please either upload them as Supporting Information files or deposit them to a stable, public repository and provide us with the relevant URLs, DOIs, or accession numbers. For a list of recommended repositories, please see <https://journals.plos.org/plosone/s/recommended-repositories>.

If there are ethical or legal restrictions on sharing a de-identified data set, please explain them in detail (e.g., data contain potentially sensitive information, data are owned by a third-party organization, etc.) and who has imposed them (e.g., an ethics committee). Please also provide contact information for a data access committee, ethics committee, or other institutional body to which data requests may be sent. If data are owned by a third party, please indicate how others may request data access.

Author response: Thank you for pointing this out. I would like to revise my Data Availability Statement as follows:

“The minimal data set is included within the manuscript and its supporting information files.”

5. PLOS ONE now requires that authors provide the original uncropped and unadjusted images underlying all blot or gel results reported in a submission’s figures or Supporting Information files. This policy and the journal’s other requirements for blot/gel reporting and figure preparation are described in detail at [https://journals.plos.org/plosone/s/figures#loc-blot-and-gel-reporting-requirements](https://journals.plos.org/plosone/s/figures#loc-blot-and-gel-reporting-requirements) and [https://journals.plos.org/plosone/s/figures#loc-preparing-figures-from-image-files](https://journals.plos.org/plosone/s/figures#loc-preparing-figures-from-image-files). When you submit your revised manuscript, please ensure that your figures adhere fully to these guidelines and provide the original underlying images for all blot or gel data reported in your submission. See the following link for instructions on providing the original image data: [https://journals.plos.org/plosone/s/figures#loc-original-images-for-blots-and-gels](https://journals.plos.org/plosone/s/figures#loc-original-images-for-blots-and-gels).

In your cover letter, please note whether your blot/gel image data are in Supporting Information or posted at a public data repository, provide the repository URL if relevant, and provide specific details as to which raw blot/gel images, if any, are not available. Email us at plosone@plos.org if you have any questions.

Author response: I have included my original uncropped and unadjusted images underlying all blot or gel results in the Supporting information file.

Additional Editor Comments (if provided):

Dear authors, before the article can go into revision, it is necessary that you upload the full blots (including molecular weights and merges) of all the gels that make up the figures. Furthermore, excessive contrast of the bands in these figures is not recommended. Please review the manuscript and resubmit.


[Note: HTML markup is below. Please do not edit.]


[NOTE: If reviewer comments were submitted as an attachment file, they will be attached to this email and accessible via the submission site. Please log into your account, locate the manuscript record, and check for the action link "View Attachments". If this link does not appear, there are no attachment files.]

While revising your submission, please upload your figure files to the Preflight Analysis and Conversion Engine (PACE) digital diagnostic tool, [https://pacev2.apexcovantage.com/](https://nam12.safelinks.protection.outlook.com/?url=https%3A%2F%2Fc05y1x9s.r.us-east-2.awstrack.me%2FL0%2Fhttps%3A%252F%252Fpacev2.apexcovantage.com%252F%2F1%2F010f01922c9ed281-4e21a2fa-e487-4379-82ed-176097abd6e1-000000%2Frpon0Y0KWCrdkW8H4cCFwAcatMQ%3D178&data=05|02|changin.oh@yale.edu|09684ef41d5e4deb562008dcdde4f431|dd8cbebb21394df8b4114e3e87abeb5c|0|0|638629224138510052|Unknown|TWFpbGZsb3d8eyJWIjoiMC4wLjAwMDAiLCJQIjoiV2luMzIiLCJBTiI6Ik1haWwiLCJXVCI6Mn0%3D|0|||&sdata=%2B0ZDlzM7JppbTUi46njQo3ffIOiMfACvA%2Fgv5xaTrUM%3D&reserved=0). PACE helps ensure that figures meet PLOS requirements. To use PACE, you must first register as a user. Registration is free. Then, login and navigate to the UPLOAD tab, where you will find detailed instructions on how to use the tool. If you encounter any issues or have any questions when using PACE, please email PLOS at [figures@plos.org](mailto:figures@plos.org). Please note that Supporting Information files do not need this step.

Thank you for your consideration of our submission. We look forward to the possibility of sharing our research with your readers.

Sincerely,

Changin Oh, DVM, PhD

Associate research scientist

Department of Genetics

Yale University School of Medicine

New Haven Connecticut 06520-8005, USA

e-mail: changin.oh@yale.edu
