# Supplementary material for: Rottlerin inhibits macropinocytosis of Porcine Reproductive and Respiratory Syndrome Virus through the PKCδ-Cofilin signaling pathway
Source: PLoS One. 2025 May 20;20(5):e0324500. doi: 10.1371/journal.pone.0324500 (PMC12091787; doi:10.1371/journal.pone.0324500)
Supplement: Attachment — Submitted filename: PONE-D-24-40479_R1.docx [file pone.0324500.s003.docx]

PLOS ONE

#### Rottlerin inhibits macropinocytosis of Porcine Reproductive and Respiratory Syndrome Virus through the PKCδ-Cofilin signaling pathway

--Manuscript Draft--

| Manuscript Number: | PONE-D-24-40479R1 |
| --- | --- |
| Article Type: | Research Article |
| Full Title: | Rottlerin inhibits macropinocytosis of Porcine Reproductive and Respiratory Syndrome Virus through the PKCδ-Cofilin signaling pathway |
| Short Title: | Rottlerin inhibits macropinocytosis of PRRSV |
| Corresponding Author: | Changin Oh, Ph.D.  Yale School of Medicine: Yale University School of Medicine New Haven, Connecticut UNITED STATES OF AMERICA |
| Keywords: | PKCδ, rottlerin, PRRSV, antivirals, macropinocytosis |
| Abstract: | Rottlerin exerts antiviral activity against various enveloped viruses, yet the mechanism by which it inhibits viral replication and the associated signaling pathways remains unclear. Here, we investigated the mechanisms for the antiviral effects of Rottlerin against Porcine Reproductive and Respiratory Syndrome Virus (PRRSV) in vitro. We demonstrate that PRRSV enters host cells via macropinocytosis. Rottlerin, a PKCδ inhibitor, partially inhibits PRRSV entry by decreasing actin polymerization. LIM domain kinase 1 (LIMK1) is essential for PRRSV replication, and PRRSV infection-induced cofilin activation, which was reversed by rottlerin treatment. Our findings suggest that a subset of PRRSV utilizes PKCδ-mediated actin dynamics to enter cells via macropinocytosis, and that rottlerin is a potential antiviral molecule targeting this entry pathway. |
| Order of Authors: | Yeonglim Kang, Ph.D. |
|  | Jong-Chul Choi, Ph.D. |
|  | Joong-Bok Lee, Ph.D. |
|  | Seung-Yong Park, Ph.D. |
|  | Changin Oh, Ph.D. |
| Opposed Reviewers: |  |
| Response to Reviewers: | PONE-D-24-40479  Rottlerin inhibits macropinocytosis of Porcine Reproductive and Respiratory Syndrome Virus through the PKCδ-Cofilin signaling pathway  PLOS ONE  Dear Dr. Gianmarco Ferrara,  Thank you for giving us the opportunity to submit a revised draft of the manuscript “Rottlerin inhibits macropinocytosis of Porcine Reproductive and Respiratory Syndrome Virus through the PKCδ-Cofilin signaling pathway” for publication in PLOS ONE. We appreciate the time and effort that you dedicated to providing feedback on our manuscript based on journal requirements. We have incorporated all of your suggestions. Those changes are highlighted within the manuscript. Please see below, in red, for a point-by point response to the editor’s comments.   1. Please ensure that your manuscript meets PLOS ONE's style requirements, including those for file naming. The PLOS ONE style templates can be found at https://journals.plos.org/plosone/s/file?id=wjVg/PLOSOne_formatting_sample_main_bo dy.pdf and https://journals.plos.org/plosone/s/file?id=ba62/PLOSOne_formatting_sample_title_aut hors_affiliations.pdf.   Author response: Thank you for pointing this out. I corrected them and highlighted.   2. Thank you for stating in your Funding Statement: |

|  | “This research was supported by the Korea Institute of Planning and Evaluation for Technology in Food, Agriculture, and Forestry (IPET) through the Agriculture, Food, and Rural Affairs Convergence Technologies Program for Educating the Creative Global Leader Program, funded by the Ministry of Agriculture, Food, and Rural Affairs (MAFRA) (320005-04).”  Please provide an amended statement that declares *all* the funding or sources of support (whether external or internal to your organization) received during this study, as detailed online in our guide for authors at <http://journals.plos.org/plosone/s/submit-> now. Please also include the statement “There was no additional external funding received for this study.” in your updated Funding Statement.  Please include your amended Funding Statement within your cover letter. We will change the online submission form on your behalf.  Author response: I revised the funding statement as follows:  “This research was supported by the Korea Institute of Planning and Evaluation for Technology in Food, Agriculture, and Forestry (IPET) through the Agriculture, Food, and Rural Affairs Convergence Technologies Program for Educating the Creative Global Leader Program, funded by the Ministry of Agriculture, Food, and Rural Affairs (MAFRA) (320005-04).” There was no additional external funding received for this study.   3. Thank you for stating the following in the Acknowledgments Section of your manuscript:   “This research was supported by the Korea Institute of Planning and Evaluation for Technology in Food, Agriculture, and Forestry (IPET) through the Agriculture, Food, and Rural Affairs Convergence Technologies Program for Educating the Creative Global Leader Program, funded by the Ministry of Agriculture, Food, and Rural Affairs (MAFRA) (320005-04). We are deeply grateful to the KU Research Center for Zoonosis for providing advice on the design and progress of this study.”  We note that you have provided additional information within the Acknowledgements Section that is not currently declared in your Funding Statement. Please note that funding information should not appear in the Acknowledgments section or other areas of your manuscript. We will only publish funding information present in the Funding Statement section of the online submission form.  Please remove any funding-related text from the manuscript and let us know how you would like to update your Funding Statement. Currently, your Funding Statement reads as follows:  “This research was supported by the Korea Institute of Planning and Evaluation for Technology in Food, Agriculture, and Forestry (IPET) through the Agriculture, Food, and Rural Affairs Convergence Technologies Program for Educating the Creative Global Leader Program, funded by the Ministry of Agriculture, Food, and Rural Affairs (MAFRA) (320005-04).”  Please include your amended statements within your cover letter; we will change the online submission form on your behalf.  Author response: Thank you for pointing this out. I revised the Acknowledgements as follows. I revised the manuscript as well.:  We are deeply grateful to the KU Research Center for Zoonosis for providing advice on the design and progress of this study.   4. We note that your Data Availability Statement is currently as follows: [All relevant data are within the manuscript and its Supporting Information files.]   Please confirm at this time whether or not your submission contains all raw data required to replicate the results of your study. Authors must share the “minimal data set” for their submission. PLOS defines the minimal data set to consist of the data required to replicate all study findings reported in the article, as well as related metadata and methods (https://journals.plos.org/plosone/s/data-availability#loc- minimal-data-set-definition).  For example, authors should submit the following data:   - The values behind the means, standard deviations and other measures reported; - The values used to build graphs; - The points extracted from images for analysis.   Authors do not need to submit their entire data set if only a portion of the data was used in the reported study.  If your submission does not contain these data, please either upload them as Supporting Information files or deposit them to a stable, public repository and provide us with the relevant URLs, DOIs, or accession numbers. For a list of recommended |
| --- | --- |

|  | repositories, please see https://journals.plos.org/plosone/s/recommended-repositories. If there are ethical or legal restrictions on sharing a de-identified data set, please explain them in detail (e.g., data contain potentially sensitive information, data are owned by a third-party organization, etc.) and who has imposed them (e.g., an ethics committee). Please also provide contact information for a data access committee, ethics committee, or other institutional body to which data requests may be sent. If data are owned by a third party, please indicate how others may request data access.  Author response: Thank you for pointing this out. I would like to revise my Data Availability Statement as follows:  “The minimal data set is included within the manuscript and its supporting information files.”  5. PLOS ONE now requires that authors provide the original uncropped and unadjusted images underlying all blot or gel results reported in a submission’s figures or Supporting Information files. This policy and the journal’s other requirements for blot/gel reporting and figure preparation are described in detail at https://journals.plos.org/plosone/s/figures#loc-blot-and-gel-reporting-requirements and https://journals.plos.org/plosone/s/figures#loc-preparing-figures-from-image-files. When you submit your revised manuscript, please ensure that your figures adhere fully to these guidelines and provide the original underlying images for all blot or gel data reported in your submission. See the following link for instructions on providing the original image data: https://journals.plos.org/plosone/s/figures#loc-original-images-for- blots-and-gels.  In your cover letter, please note whether your blot/gel image data are in Supporting Information or posted at a public data repository, provide the repository URL if relevant, and provide specific details as to which raw blot/gel images, if any, are not available.  Email us at [plosone@plos.org](mailto:plosone@plos.org) if you have any questions.  Author response: I have included my original uncropped and unadjusted images underlying all blot or gel results in the Supporting information file.  Additional Editor Comments (if provided):  Dear authors, before the article can go into revision, it is necessary that you upload the full blots (including molecular weights and merges) of all the gels that make up the figures. Furthermore, excessive contrast of the bands in these figures is not recommended. Please review the manuscript and resubmit.  [Note: HTML markup is below. Please do not edit.]  [NOTE: If reviewer comments were submitted as an attachment file, they will be attached to this email and accessible via the submission site. Please log into your account, locate the manuscript record, and check for the action link "View Attachments". If this link does not appear, there are no attachment files.]  While revising your submission, please upload your figure files to the Preflight Analysis and Conversion Engine (PACE) digital diagnostic tool, https://pacev2.apexcovantage.com/. PACE helps ensure that figures meet PLOS requirements. To use PACE, you must first register as a user. Registration is free.  Then, login and navigate to the UPLOAD tab, where you will find detailed instructions on how to use the tool. If you encounter any issues or have any questions when using PACE, please email PLOS at [figures@plos.org.](mailto:figures@plos.org) Please note that Supporting Information files do not need this step.  Thank you for your consideration of our submission. We look forward to the possibility of sharing our research with your readers.  Sincerely,  Changin Oh, DVM, PhD Associate research scientist |
| --- | --- |

|  | Department of Genetics  Yale University School of Medicine  New Haven Connecticut 06520-8005, USA e-mail: [changin.oh@yale.edu](mailto:changin.oh@yale.edu) |
| --- | --- |
| Additional Information: |  |
| Question | Response |
| Financial Disclosure  Enter a financial disclosure statement that describes the sources of funding for the work included in this submission. Review the [submission guidelines](http://journals.plos.org/plosone/s/submission-guidelines#loc-financial-disclosure-statement) for detailed requirements. View published research articles from [*PLOS ONE*](https://journals.plos.org/plosone/) for specific examples.  This statement is required for submission and will appear in the published article if the submission is accepted. Please make sure it is accurate. | Yes |
| Funded studies  Enter a statement with the following details:   - Initials of the authors who received each award - Grant numbers awarded to each author - The full name of each funder - URL of each funder website - Did the sponsors or funders play any role in the study design, data collection and analysis, decision to publish, or preparation of the manuscript? |  |
| Did you receive funding for this work? |  |
| Please add funding details.  as follow-up to "Financial Disclosure  Enter a financial disclosure statement that describes the sources of funding for the work included in this submission. Review the [submission guidelines](http://journals.plos.org/plosone/s/submission-guidelines#loc-financial-disclosure-statement) for detailed requirements. View published research articles from [*PLOS ONE*](https://journals.plos.org/plosone/) for specific examples.  This statement is required for submission and will appear in the published article if | This research was supported by the Korea Institute of Planning and Evaluation for Technology in Food, Agriculture, and Forestry (IPET) through the Agriculture, Food, and Rural Affairs Convergence Technologies Program for Educating the Creative Global Leader Program, funded by the Ministry of Agriculture, Food, and Rural Affairs (MAFRA) (320005-04).” There was no additional external funding received for this study. |

| the submission is accepted. Please make sure it is accurate. |  |
| --- | --- |
| Funded studies  Enter a statement with the following details:   - Initials of the authors who received each award - Grant numbers awarded to each author - The full name of each funder - URL of each funder website - Did the sponsors or funders play any role in the study design, data collection and analysis, decision to publish, or preparation of the manuscript? |  |
| Did you receive funding for this work?" |  |
| Please select the country of your main research funder (please select carefully as in some cases this is used in fee calculation).  as follow-up to "Financial Disclosure  Enter a financial disclosure statement that describes the sources of funding for the work included in this submission. Review the [submission guidelines](http://journals.plos.org/plosone/s/submission-guidelines#loc-financial-disclosure-statement) for detailed requirements. View published research articles from [*PLOS ONE*](https://journals.plos.org/plosone/) for specific examples.  This statement is required for submission and will appear in the published article if the submission is accepted. Please make sure it is accurate. | KOREA, REPUBLIC OF - KR |
| Funded studies  Enter a statement with the following details:   - Initials of the authors who received each award - Grant numbers awarded to each author - The full name of each funder - URL of each funder website - Did the sponsors or funders play any role in the study design, data collection and analysis, decision to publish, or preparation of the manuscript? |  |
|  |  |

| Did you receive funding for this work?" |  |
| --- | --- |
| Competing Interests  Use the instructions below to enter a competing interest statement for this submission. On behalf of all authors, disclose any [competing interests](http://journals.plos.org/plosone/s/competing-interests) that could be perceived to bias this work—acknowledging all financial support and any other relevant financial or non- financial competing interests.  This statement is required for submission and will appear in the published article if the submission is accepted. Please make sure it is accurate and that any funding sources listed in your Funding Information later in the submission form are also declared in your Financial Disclosure statement.  View published research articles from  [*PLOS ONE*](http://journals.plos.org/plosone/) for specific examples. | The authors have declared that no competing interests exist. |
| NO authors have competing interests  Enter: *The authors have declared that no competing interests exist.*  Authors with competing interests  Enter competing interest details beginning with this statement:  *I have read the journal's policy and the authors of this manuscript have the following competing interests: [insert competing interests here]* |  |
| * typeset |  |
| Ethics Statement  Enter an ethics statement for this submission. This statement is required if the study involved:   - Human participants - Human specimens or tissue | N/A |

| - Vertebrate animals or cephalopods - Vertebrate embryos or tissues - Field research   Write "N/A" if the submission does not require an ethics statement.  General guidance is provided below. Consult the [submission guidelines](http://journals.plos.org/plosone/s/submission-guidelines#loc-guidelines-for-specific-study-types) for detailed instructions. Make sure that all information entered here is included in the Methods section of the manuscript. |  |
| --- | --- |

| Format for specific study types  Human Subject Research (involving human participants and/or tissue)   - Give the name of the institutional review board or ethics committee that approved the study - Include the approval number and/or a statement indicating approval of this research - Indicate the form of consent obtained (written/oral) or the reason that consent was not obtained (e.g. the data were analyzed anonymously)   Animal Research (involving vertebrate animals, embryos or tissues)   - Provide the name of the Institutional Animal Care and Use Committee (IACUC) or other relevant ethics board that reviewed the study protocol, and indicate whether they approved this research or granted a formal waiver of ethical approval - Include an approval number if one was obtained - If the study involved *non-human primates*, add *additional details* about animal welfare and steps taken to ameliorate suffering - If anesthesia, euthanasia, or any kind of animal sacrifice is part of the study, include briefly which substances and/or methods were applied   Field Research  Include the following details if this study involves the collection of plant, animal, or other materials from a natural setting:   - Field permit number - Name of the institution or relevant body that granted permission |  |
| --- | --- |
|  |  |
| Data Availability  Authors are required to make all data underlying the findings described fully available, without restriction, and from the time of publication. PLOS allows rare exceptions to address legal and ethical concerns. See the [PLOS Data Policy](http://journals.plos.org/plosone/s/data-availability) and [FAQ](http://journals.plos.org/plosone/s/data-availability#loc-faqs-for-data-policy) for detailed information. | Yes - all data are fully available without restriction |

| A Data Availability Statement describing where the data can be found is required at submission. Your answers to this question constitute the Data Availability Statement and will be published in the article, if accepted. |  |
| --- | --- |
| Important: Stating ‘data available on request from the author’ is not sufficient. If your data are only available upon request, select ‘No’ for the first question and explain your exceptional situation in the text box. |  |
| Do the authors confirm that all data underlying the findings described in their manuscript are fully available without restriction? |  |
| Describe where the data may be found in full sentences. If you are copying our sample text, replace any instances of XXX with the appropriate details.   - If the data are held or will be held in a public repository, include URLs, accession numbers or DOIs. If this information will only be available after acceptance, indicate this by ticking the box below. For example: *All XXX files are available from the XXX database (accession number(s) XXX, XXX.*). - If the data are all contained within the manuscript and/or Supporting Information files, enter the following: *All relevant data are within the manuscript and its Supporting Information files.* - If neither of these applies but you are able to provide details of access elsewhere, with or without limitations, please do so. For example:   *Data cannot be shared publicly because of [XXX]. Data are available from the XXX Institutional Data Access / Ethics Committee (contact via XXX) for researchers who meet the criteria for access to confidential data.*  *The data underlying the results presented in the study are available from (include the name of the third party* | The minimal data set is included within the manuscript and its supporting information files. |

| *and contact information or URL).*   - This text is appropriate if the data are owned by a third party and authors do not have permission to share the data.   * typeset |  |
| --- | --- |
| Additional data availability information: |  |

Cover Letter

September 12^th^ , 2024

*PLOS ONE*

To the Editor:

We wish to submit an original research paper titled **“Rottlerin inhibits macropinocytosis of Porcine Reproductive and Respiratory Syndrome Virus through the PKCδ- Cofilin signaling pathway”** for publication in *PLOS ONE*. The paper was co-authored by Yeonglim Kang, Joong-Bok Lee, and Seung-Yong Park.

This study builds on our previous publication, which demonstrated that rottlerin showed antiviral activity against the porcine reproductive and respiratory syndrome virus (PRRSV) in vitro and in vivo by blocking its initial entry. In the current study, we demonstrated that PRRSV induced macropinocytosis on infected cells and rottlerin blocks it by decreasing actin rearrangement, with PRRSV-mediated activation of cofilin and LIM domain kinase 1 (LIMK1) occurring through the PKCδ signaling pathway.

We believe this work will be of great interest to readers in veterinary medicine because, as PRRSV continues to be a major challenge in the field, and effective control methods are urgently needed. Additionally, our findings may appeal to a broader reader in your journal, as they provide insight into a potential mechanism of action for rottlerin against various types of viruses.

This manuscript has not been published or presented elsewhere in part or in entirety and is not under consideration by any other journal. The study design was approved by the appropriate ethics review board. We have read and understood your journal’s policies, and we believe that neither the manuscript nor the study violates any of these. There are no conflicts of interest to declare.

Thank you for your consideration of our submission. We look forward to the possibility of sharing our research with your readers.

Sincerely,

Changin Oh, DVM, PhD Associate research scientist Department of Genetics

Yale University School of Medicine

New Haven Connecticut 06520-8005, USA e-mail: [changin.oh@yale.edu](mailto:changin.oh@yale.edu)

<https://www2.cloud.editorialmanager.com/pone/download.aspx?id=38216029&guid=b7052acc-3d7f-4f5a-b8c9-cd121ccabb62&scheme=1>Manuscript	[Click here to](https://www2.cloud.editorialmanager.com/pone/download.aspx?id=38216029&guid=b7052acc-3d7f-4f5a-b8c9-cd121ccabb62&scheme=1)

[access/download;Manuscript;Rotterlin_PRRSV_macropinocytosi](https://www2.cloud.editorialmanager.com/pone/download.aspx?id=38216029&guid=b7052acc-3d7f-4f5a-b8c9-cd121ccabb62&scheme=1)

1 **Rottlerin inhibits macropinocytosis of Porcine Reproductive and**
2 **Respiratory Syndrome Virus through the PKCδ-Cofilin signaling**
3 **pathway**

4

5	Yeonglim Kang^a^, Jong-Chul Choi^a^, Joong-Bok Lee^a^, Seung-Yong Park^a^, and Changin Oh^b*^

6

7 a*Laboratory of Infectious Diseases, College of Veterinary Medicine, Konkuk University, Seoul*
8 *05029, Republic of Korea*
9 b*Department of Genetics, Yale University School of Medicine, New Haven, CT 06520-8005, USA*

10

11 ***Corresponding author:** Changin Oh
12 Department of Genetics, Yale University School of Medicine, New Haven, CT 06520-8005, USA
13 E-mail: [changin.oh@yale.edu](mailto:changin.oh@yale.edu)

### 14 Abstract

15 Rottlerin exerts antiviral activity against various enveloped viruses, yet the mechanism by which
16 it inhibits viral replication and the associated signaling pathways remains unclear. Here, we
17 investigated the mechanisms for the antiviral effects of Rottlerin against Porcine Reproductive and
18 Respiratory Syndrome Virus (PRRSV) in vitro. We demonstrate that PRRSV enters host cells via
19 macropinocytosis. Rottlerin, a PKCδ inhibitor, partially inhibits PRRSV entry by decreasing actin
20 polymerization. LIM domain kinase 1 (LIMK1) is essential for PRRSV replication, and PRRSV
21 infection-induced cofilin activation, which was reversed by rottlerin treatment. Our findings
22 suggest that a subset of PRRSV utilizes PKCδ-mediated actin dynamics to enter cells via
23 macropinocytosis, and that rottlerin is a potential antiviral molecule targeting this entry pathway.

24

###### 25 Keywords

26 PKCδ, rottlerin, PRRSV, antivirals, macropinocytosis

27

### 28 Introduction

29 Macropinocytosis is an endocytic process that leads to the internalization of fluids, extracellular
30 particles, and membranes into large vacuoles (1). Activation of actin and microfilaments induces
31 macropinocytosis through the formation of lamellipodia, membrane ruffles, and blebs on the
32 cellular periphery (2). Various viral families, such as human immunodeficiency virus (HIV),
33 vaccinia virus, and adenovirus, and various types of bacteria take advantage of macropinocytosis
34 as a direct means of cellular internalization or an indirect mechanism to assist the penetration of
35 particles that have entered by other forms of endocytosis (2). Thus, blocking macropinocytosis
36 may be a potential strategy for controlling broad-spectrum viral infections.
37 Porcine reproductive and respiratory syndrome virus (PRRSV) is an enveloped positive-sense
38 ssRNA virus belonging to the family Arteriviridae and order Nidovirales (3, 4). PRRSV infection
39 causes reproductive impairments in sows and respiratory diseases in pigs of all ages, resulting in
40 a critical economic impact on the global swine industry (5, 6). It has been reported that PRRSV
41 enters into host cells via low pH-dependent, clathrin-mediated endocytosis, utilizing CD163 as an
42 essential receptor (7-9). A recent paper claims that PRRSV uses macropinocytosis as an alternative
43 entry route (10). However, Additional evidence and detailed mechanisms underlying PRRSV-
44 induced macropinocytosis are necessary to support this novel finding. We recently demonstrated
45 that rottlerin blocks PRRSV endocytosis by inhibiting PRRSV-induced protein kinase C delta
46 (PKCδ) phosphorylation (11), suggesting that inhibition of PKCδ phosphorylation suppresses one
47 or more PRRSV endocytosis routes. Thus, we investigated the antiviral mechanisms of rottlerin
48 against PRRSV, especially how rottlerin blocks PRRSV endocytosis.
50 Here, we demonstrated that PRRSV enters MARC-145 cells via macropinocytosis during infection,
51 which is inhibited by rottlerin treatment, by decreasing actin polymerization. PRRSV
52 macropinocytosis is mediated by the PKCδ-cofilin pathway and LIM domain kinase 1 (LIMK1).
53 The results of the present study provide a possible explanation for the antiviral effect of rottlerin
54 on other viruses and contribute to an understanding of the PRRSV entry mechanism.

54

### 55 Methods

###### 56 Viruses

57 The PRRSV FL12 strain used in this study was rescued from the pCMV-FL12/24 plasmid that was
58 kindly provided by Professor Won-Il Kim (12). Briefly, semi-confluent MARC-145 cells were
59 transfected with pCMV-FL12/24 and the Via Fect reagent (Promega, Madison, WI, USA) in a 1:3
60 ratio. After 72 hours of transfection, cell supernatants were harvested and passaged into MARC-
61 145 cells. Viral titers were measured using quantitative reverse transcription–polymerase chain
62 reaction (qRT-PCR) and titration. Viral stocks were stored at −80 ℃. Viruses were concentrated
63 by centrifugation using a Beckman SW-41 Ti rotor at 160,000 × *g* at 4 ℃ for 4 hours through a
64 discontinuous sucrose gradient consisting of 1 mL of 25%, 35%, 45%, 55%, and 65% sucrose. The
65 35%–45% fraction was dialyzed and titrated for future experiments.

66

###### 67 Immunofluorescence assay and confocal microscopy

68 Approximately 2.4 × 10^4^ MARC-145 cells per well were seeded on an eight-chamber slide. On
69 the following day, MARC-145 cells were preincubated with rottlerin (5 μM) or 5-[*N*-ethyl-*N*-
70 isopropyl] amiloride (EIPA) (50 μM) (Tocris, Bristol, UK) at 37 °C for 2 hours or left untreated.
71 For the viral endocytosis assay, cells were infected with PRRSV FL12 at a multiplicity of infection
72 (MOI) of 50 for 1 hour. Cells were washed and treated with proteinase K to remove extracellular
73 viruses for 45 minutes at 4°C. For the dextran uptake assay, cells were inoculated with CF®640R
74 dextran (10,000 MW) (Biotium, Fremont, CA, USA) with or without PRRSV FL12 (MOI = 50),
75 incubated for 1 hour, and washed with 3 M sodium acetate buffer (pH 5.2). The cells were fixed
76 with 4% paraformaldehyde for 15 minutes and permeabilized with Tris-buffered saline (TBS)
77 containing 0.2% Triton X-100 at room temperature (RT) for 10 minutes. The cells were then
78 probed as previously described (13) with minor modifications. CF®640R phalloidin (Biotium,
79 Fremont, CA, USA) was used to label the F-actin. An anti-PRRSV nucleocapsid (N) monoclonal
80 antibody (1:200) (Median Diagnostics, Chuncheon, Korea) was used to label the viral particles.
81 The cells were scanned using an EVOS FL Cell Imaging System (Thermo Fisher Scientific,
82 Waltham, MA, USA) or an LSM 900 with Airyscan 2 (Zeiss, Oberkochen, Germany). The images
83 were processed, and the corrected total cell fluorescence was calculated using ImageJ software
84 (NIH, Bethesda, MD, USA).

85

###### 86 RNA interference

87 To silence PKCδ, LIMK1, and cofilin expression, MARC-145 cells were transfected with small
88 interfering RNAs (siRNAs) targeting PKCδ, LIMK1, and cofilin, respectively (14) using
89 Lipofectamine 3000 (Thermo Fisher Scientific ) according to the manufacturer’s instructions, and
90 the cells were incubated at 37 °C for 24 hours.

91

###### 92 Western blot

93 To test the effects of rottlerin on the phosphorylation of LIMK1 and cofilin, confluent MARC-145
94 cells were treated with rottlerin (5 μM) or EIPA (50 μM), ~~respectively~~, for 2 hours at 37 ℃. The
95 cells were inoculated with PRRSV FL12 (MOI = 1) and incubated for the indicated times. Cells
96 were lysed using mammalian protein extraction reagent (Thermo Fisher Scientific) with a Halt
97 protease phosphatase inhibitor cocktail (Thermo Fisher Scientific). Cell lysates were resolved
98 using sodium dodecyl sulfate-polyacrylamide gel electrophoresis (Bolt 4%–12% Bis-Tris gel;
99 Invitrogen, Waltham, MA, USA) and transferred onto nitrocellulose membranes (Komabiotech,
100 Seoul, Korea). The membranes were blocked with 5% skim milk in TBS with Tween 20 (TBS-T)
101 for 30 minutes at RT and probed with rabbit anti-cofilin (P-cofilin, Ser3) monoclonal antibody
102 (1:1,000) (Cell Signaling Technology, Danvers, MA, USA), mouse anti-cofilin monoclonal
103 antibody–horseradish peroxidase (HRP) (1:1,000) (Santa Cruz Biotechnology, Dallas, TX, USA),
104 rabbit anti-LIMK1/2 (P-LIMK1, Thr508/505) polyclonal antibody (1:1,000) (BioVision, Waltham,
105 MA, USA), rabbit anti-LIMK1 polyclonal antibody (1:1,000) (Cell Signaling Technology), or
106 mouse anti-β-actin monoclonal antibody-HRP (1:50,000) (Abcam, Cambridge, UK) overnight at
107 4 ℃. After five washes with TBS-T, the membranes were incubated with HRP-conjugated anti-
108 rabbit IgG (Komabiotech) (1:5,000) for 1 hour at RT. Proteins were visualized using Clarity
109 Western ECL Substrate (Bio-Rad, Hercules, CA, USA) and detected using FUSION Solo S (Vilber,
110 Collégien, France). The density of the protein bands was measured using ImageJ software (NIH)
111 after subtracting the density of the β-actin bands.

112

###### 113 qRT-PCR and virus titration

114 Viral RNA was extracted from the supernatants of PRRSV-infected cells using an RNeasy Mini
115 Kit (QIAGEN, Hilden, Germany), according to the manufacturer's recommendations. Viral RNA
116 levels were quantified using the RNA UltraSense One-Step Quantitative RT-PCR System
117 (Invitrogen) with the LightCycler 96 System (Roche, Basel, Switzerland), as previously described
118 (15). The measured cycle threshold (CT) values were converted to RNA copy numbers and tissue
119 culture infective dose 50% (TCID50) equivalent/mL using a standard curve generated from CT
120 values plotted against the corresponding TCID50/mL. The viral titer (TCID50/mL) of the collected
121 samples was measured using the Reed–Muench method, as previously described (16).

122

###### 123 Statistical analyses

124 All data were analyzed using IBM SPSS Statistics 25 (IBM Corp., Armonk, NY, USA) and
125 GraphPad Prism 6 (GraphPad Software, San Diego, CA, USA) using a nonparametric model
126 (Kruskal–Wallis followed by Mann–Whitney). All data are presented as the mean ± standard
127 deviation, and *, *p*-value <0.05; **, *p*-value <0.01; ***, *p*-value <0.001 were considered
128 significant.

129

### 130 Results

###### 131 Rottlerin inhibits PRRSV endocytosis in MARC-145 cells

132 We previously reported that rottlerin inhibits PRRSV endocytosis(11), but the detailed mechanism
133 of its antiviral activity and involvement of other PKCδ related cellular molecules is unknown. To
134 investigate the role of the PKCδ and elucidate the antiviral mechanism of rottlerin during PRRSV
135 infection, we examined PRRSV replication in the presence of rottlerin in MARC-145 cells. The
136 cells were pre-treated with rottlerin (5 μM) or EIPA (50 μM), or subjected to PKCδ silencing,
137 followed by infection with PRRSV FL12 (MOI= 1). Subsequently, viral replication was evaluated
138 using three distinct parameters at 48 hours post-infection (hpi): PRRSV RNA synthesis, PRRSV
139 nucleocapsid (N) protein expression, and infectious virus production. Consistent with our previous
140 report, both rottlerin and siRNA treatment significantly inhibited viral RNA synthesis (Fig. 1A),
141 expression of the N protein (Fig. 1B), and production of infectious progeny viruses (Fig. 1C).
142 To further determine whether rottlerin blocks PRRSV endocytosis, we used immunofluorescence
143 to observe the localization of PRRSV particles in infected MARC-145 cells after treatment with
144 rottlerin or RIPA. The cells were washed with sodium acetate buffer (pH 5.2) to eliminate the
145 extracellular viruses. As expected, confocal microscopy images of dimethyl sulfoxide (DMSO)-
146 treated cells showed localization of PRRSV to the cytoplasm with a puncta shape (Fig. 1D, left),
147 whereas rottlerin-treated cells showed the absence of a signal in the cytoplasm (Fig. 1D, middle),
148 similar to EIPA treated cells (Fig. 1D, right). This indicates that rottlerin treatment impaired
149 PRRSV endocytosis. These results are consistent with our previous findings showing that rottlerin
150 treatment suppressed PRRSV replication in cells and pigs by inhibiting PRRSV endocytosis (11).

###### 151 Rottlerin inhibited macropinocytosis of PRRSV by decreasing actin polymerization

152 Due to the inhibition of PRRSV replication and endocytosis by EIPA treatment (Fig. 1), a selective
153 macropinocytosis	inhibitor	(17),	we	investigated	whether	PRRSV	entry	occurs	via
154 macropinocytosis and if rottlerin inhibits PRRSV macropinocytosis. We co-incubated MARC-145
155 cells for an hour with fluorescence-labeled CF®640R dextran (10,000 MW), which is known to
156 enter cells via macropinocytosis (18, 19) (Fig. 2A). In DMSO-treated cells, dextran signals were
157 observed in the cytoplasm, suggesting macropinocytosis of dextran (Fig. 2A, top). In contrast,
158 rottlerin or EIPA-treated cells showed reduced dextran signals around the nucleus (Fig. 2A, middle
159 and bottom), indicating that rottlerin also inhibits macropinocytosis.
160 Next, PRRSV and dextran were co-inoculated into DMSO, rottlerin, or EIPA-treated cells. In
161 DMSO-treated cells, signals for both dextran and PRRSV were observed around the nucleus with
162 some colocalization (Fig. 2B, top row). This suggests that during the endocytosis of the virus and
163 dextran, some of them enter together through macropinocytosis. In contrast, either rottlerin or EIPA
164 treatment significantly decreased the number of both signals in cells with significantly less co-
165 localization between them (Fig. 2C, Pearson’s coefficient: DMSO, 0.43 vs. rottlerin, 0.07). These
166 results suggest that rottlerin inhibited PRRSV macropinocytosis in MARC-145 cells.
167 Among the several characteristics of macropinocytosis, actin polymerization is a key factor for
168 macropinocytosis-specific uptake (10, 20). Therefore, we tested whether rottlerin reduced actin
169 polymerization during PRRSV macropinocytosis similar to EIPA. Actin dynamics were observed
170 using fluorescently labeled phalloidin, a specific ligand of F-actin. DMSO-treated cells after
171 PRRSV infection showed multiple pseudopods projecting from the plasma membrane into the
172 outer space (white arrow) (Fig. 2D, top row). Interestingly, rottlerin or EIPA treatment caused the
173 disappearance of these projections (Fig. 2D, middle and bottom rows). Quantitative analysis
174 showed that cells with proper projections were significantly decreased when treated with rottlerin
175 (*p*-value < 0.05) and EIPA (*p*-value < 0.01) compared with the DMSO-treated cells (Fig. 2E),
176 confirming that rottlerin inhibited PRRSV macropinocytosis by reducing actin polymerization.

177

###### 178 Rottlerin inhibits PRRSV endocytosis through the PKCδ-cofilin signaling pathway

179 PKCδ regulates actin dynamics during macropinocytosis via downstream effectors such as cofilin,
180 slingshot phosphatase homolog 1, and LIMK1(21). Cofilin regulates the dynamics of the actin
181 cytoskeleton by binding to an actin filament(22, 23). LIMK1 directly phosphorylates cofilin at Ser-
182 3, which inactivates it (21, 24, 25). To evaluate the involvement of cofilin and LIMK1 in PRRSV
183 macropinocytosis, cofilin- or LIMK1-silenced MARC-145 cells were used to measure PRRSV
184 replication. Transfection of siRNAs targeting cofilin or LIMK1 effectively knocked down the
185 expression of each protein in the cells (Fig. 3A). Interestingly silencing LIMK1 resulted in
186 decreased expression of cofilin.
187 Next, we investigated the effect of cofilin or LIMK1 silencing on PRRSV replication by measuring
188 viral titer and expression of the N protein in siRNA-treated cells after PRRSV infection. The N
189 protein blot showed that silencing LIMK1 significantly inhibited N protein synthesis with the
190 relative intensity of the band being 0.36 compared to treatment with siNC (Fig. 3A, Relative N
191 protein intensity:100). Silencing Cofilin resulted in an intensity of 0.84 for the N protein band, but
192 this effect was not as significant as that observed with LIMK1 silencing. Consistent with the
193 western blot results, the silencing of LIMK1 notably reduced the viral titer by 11-fold compared
194 to siNC-treated cells (Fig. 3B), whereas cofilin silencing did not significantly impact the viral titer.
195 These results suggest that LIMK1 serves as a host dependency factor essential for PRRSV
196 infection.
197 To further investigate how macropinocytosis is regulated by rottlerin following PRRSV infection,
198 we monitored the activity of cofilin or LIMK1 in PRRSV-infected cells. Since the activities of
199 LIMK1 and cofilin are regulated by phosphorylation, we examined the levels of phosphorylated
200 LIMK1 and cofilin (P-LIMK1 and p-cofilin) as well as total LIMK1 and cofilin using immunoblots.
201 As shown in Fig. 3C and D, the levels of P-LIMK1 after PRRSV infection transiently increased
202 immediately after PRRSV infection (15 mpi), slightly dephosphorylated (30 mpi), and then re-
203 phosphorylated at a later time point (120 mpi). In contrast, rottlerin treatment rapidly attenuated
204 the virus-induced P-LIMK1, and the P-LIMK1 levels in rottlerin-treated cells were returned
205 similar to Mock-treated cells at 30, and 120 mpi (Fig. 3C and D). Similar levels of total LIMK1
206 after DMSO or rottlerin treatment suggest that the changes in P- LIMK1 were not due to a change
207 in total LIMK1. These results suggest that PRRSV infection mediates transient activation of
208 LIMK1 at an early stage of infection through PKCδ, which was rapidly attenuated by rottlerin.
209 PRRSV infection gradually decreased P-cofilin levels in DMSO-treated cells, and a low level of
210 P-cofilin was maintained up to 120 mpi (Fig. 3E and F). In contrast, rottlerin treatment led to
211 increase P- cofilin. The levels of P-cofilin in rottlerin-treated cells began to increase at 15 mpi and
212 continued to rise until 120 mpi (Fig. 3E and F). Quantitative analysis of the blot bands showed that
213 rottlerin treatment significantly increased P-cofilin levels at 90 and 120 mpi compared with DMSO
214 treatment (Fig. 3F). These results demonstrate that PRRSV infection decreases P-cofilin levels,
215 whereas rottlerin treatment reverses this effect. Overall, the findings suggest that rottlerin regulates
216 LIMK1 and cofilin signaling pathways, which may contribute to the mechanism by which rottlerin
217 inhibits PRRSV-induced macropinocytosis**.**

### 218 Discussion

219 In this study, our microscopic and biochemical analyses demonstrated that rottlerin treatment
220 inhibited PRRSV replication by decreasing viral endocytosis in vitro, which resulted in the
221 suppression of viral RNA synthesis, N protein expression, and infectious virus production (Fig. 1).
222 We also showed that PRRSV entered MARC-145 cells via macropinocytosis, and rottlerin blocked
223 it by decreasing actin polymerization (Fig. 2). LIMK1 acts as a host dependency factor crucial for
224 PRRSV infection. Rottlerin reversed PRRSV-induced activation of cofilin (Fig. 3). These findings
225 provide a possible explanation for one of the antiviral mechanisms of rottlerin at the early stages
226 of virus infection, i.e., rottlerin blocks macropinocytosis of viruses by decreasing actin
227 polymerization via the PKCδ-cofilin signaling pathway. In the case of HIV1 and Zika virus,
228 rottlerin inhibits an early stage of their replication (26, 27) and they are known to enter cells via
229 actin-related entry pathways, indicating that rottlerin could inhibit their entry through a similar
230 mechanism (28-30).
231 Our results demonstrated that macropinocytosis serves as an alternative route for PRRSV to
232 infect host cells. Our findings match with a recent study that PRRSV infects host cells through
233 macropinocytosis, involving T-cell immunoglobulin and mucin domain proteins as well as CD163
234 (10). We further hypothesize that PRRSV possibly enters cells via at least two distinct pathways:
235 clathrin-mediated endocytosis and macropinocytosis. Few in vitro assay results support the
236 hypothesis that a single treatment with chlorpromazine, a clathrin lattice polymerization inhibitor,
237 or rottlerin partially blocked PRRSV replication (Fig. 1C), but the combination of both inhibitors
238 almost completely inhibited PRRSV replication (10). Understanding the potential PRRSV entry
239 routes is crucial for the clinical application of antiviral drugs. To achieve an effective anti-PRRSV
240 treatment, a combination of inhibitors targeting both entry routes, rather than a single treatment
241 with either route inhibitor, is necessary.
242 Our results provide insights into how PRRSV induces macropinocytosis through the PKCδ-
243 LIMK1-cofilin signaling pathway to infect host cells. Although macropinocytosis signaling
244 mechanisms have been extensively studied over the last decade (2, 21), the PRRSV-mediated
245 signaling pathway of macropinocytosis remains unclear. Generally, to induce macropinocytosis by
246 cofilin and LIMK1, the binding of the active form of cofilin (dephosphorylated at Ser3)
247 depolymerizes actin by severing the actin filaments, which finally induces lamellipodium
248 formation and macropinocytosis(31). However, filament severing also results in actin
249 polymerization, depending on the relative amount of actin monomers and free barb ends (32). LIM
250 kinases directly phosphorylate cofilin, which allows the precise regulation of actin dynamics (33).
251 LIMK1 is known to not only phosphorylate cofilin but also directly bind F-actin to induce actin
252 depolymerization and membrane ruffling (34). In the case of PRRSV, we propose that viral
253 infection activates cofilin to increase lamellipodium formation for macropinocytosis, which
254 increases viral uptake from the extracellular space (21). Rottlerin treatment inhibits this activation
255 by blocking upstream PKCδ(11). Our microscopic data is in line with this model (Fig. 2).
256 Our results regarding LIMK1 phosphorylation cannot be fully explained by the standard cofilin-
257 LIMK1 pathway. In our study, PRRSV infection induced LIMK1 phosphorylation (Fig. 3C and
258 D), which is contrary to what would be expected based on our microscopic and cofilin
259 phosphorylation results. Typically, phosphorylated LIMK1 adds a phosphate group to cofilin,
260 thereby inactivating cofilin and stabilizing actin filaments. Considering all our results, LIMK1
261 may play an additional role in this virus entry process. It is possible that LIMK1 not only
262 phosphorylates cofilin but also precisely regulates this pathway. Previous research shows LIMK1
263 is involved in the microtubule destabilization(35), suggesting LIMK1 may directly coordinate
264 microtubules and actin cytoskeleton. Another research also supports this notion, indicating that
265 pharmacological inhibition of LIMK1 does not stimulate macropinocytosis(21). Even if LIMK1
266 did not follow the standard pathway, it appears that LIMK1 is a key regulator employed by PRRSV
267 to control macropinocytosis. These results are consistent with previous studies showing that
268 PRRSV replication requires PKCδ (36). HIV binding to CD4+ T cells initiates transient activation
269 of LIMK and actin polymerization (37). Small-molecule inhibitors of LIM kinases have been
270 reported to inhibit HIV1 (38). Further research is needed to study the mechanisms underlying
271 virus-induced macropinocytosis.
272 Rottlerin is a secondary metabolite isolated from *Mallotus philippinensis*, which has been
273 traditionally used to treat tapeworm, scabies, and herpetic ringworm infections in India for several
274 centuries (39). Rottlerin is a selective inhibitor of PKCδ (40, 41), but recent studies have reported
275 that rottlerin inhibits multiple kinases (42-44). In our study, PKCδ inhibition of rottlerin is highly
276 likely to be a major blocking mechanism of PRRSV macropinocytosis, rather than other
277 mechanisms, because silencing of PKCδ decreased PRRSV replication (Fig. 1), and we have
278 previously shown that rottlerin treatment dephosphorylates PKCδ induced by PRRSV infection
279 (11). Interestingly, several other studies have demonstrated the PKCδ-independent antiviral effects
280 of rottlerin (43, 44). Ojha et al. recently showed that rottlerin inhibits La Crosse virus trafficking
281 from the Golgi apparatus to the trans-Golgi vesicles, which may be related to decreased cellular
282 ATP levels induced by rottlerin (43, 45). These results suggest that rottlerin can block various steps
283 of viral replication, thus strengthening its antiviral potency. Thus, an intensive mechanistic study
284 of the antiviral effects of rottlerin against various viruses is required.
285 In conclusion, the current study demonstrates that PRRSV utilizes macropinocytosis to infect host
286 cells. Rottlerin blocks macropinocytosis of PRRSV related to PKCδ-cofilin signaling pathways by
287 reducing actin polymerization. Our results suggest that rottlerin is a potential antiviral drug
288 targeting macropinocytosis and its associated signaling molecules.

289

290

###### 291 Clinical Perspectives

292 **Point 1.** Rottlerin inhibited PRRSV replication by reducing viral endocytosis.
293 **Point 2.** Rottlerin suppresses PRRSV-induced macropinocytosis and actin polymerization through
294 the PKCδ-cofilin signaling pathway.
295 **Point 3.** Rottlerin is a potential broad-spectrum antiviral drug targeting macropinocytosis.

296

###### 297 Data availability statement

298 All data needed to evaluate the conclusions in the paper are present in the paper.

###### 299 Author Contribution

300 Y.K. and C.O. designed research; Y.K., I.C., and J.C. performed research; Y.K., J.C., and C.O.
301 analyzed data; Y.K., J.L., S.P., and C.O. wrote the paper.

###### 302 Declaration of competing interest

303 The authors declare no competing interests.

304

305

###### 306 Acknowledgment

307 This research was supported by the Korea Institute of Planning and Evaluation for Technology in
308 Food, Agriculture, and Forestry (IPET) through the Agriculture, Food, and Rural Affairs
309 Convergence Technologies Program for Educating the Creative Global Leader Program, funded
310 by the Ministry of Agriculture, Food, and Rural Affairs (MAFRA) (320005-04). We are deeply
311 grateful to the KU Research Center for Zoonosis for providing advice on the design and progress
312 of this study.

313

### 314 References

315 1.	Zeineddine R, Yerbury JJ. The role of macropinocytosis in the propagation of protein
316 aggregation associated with neurodegenerative diseases. Front Physiol. 2015;6:277.
317 2.	Mercer J, Helenius A. Virus entry by macropinocytosis. Nat Cell Biol. 2009;11(5):510-20.
318 3.	Cavanagh D. Nidovirales: a new order comprising Coronaviridae and Arteriviridae. Arch Virol.
319 1997;142(3):629-33.
320 4.	Conzelmann KK, Visser N, Van Woensel P, Thiel HJ. Molecular characterization of porcine
321 reproductive and respiratory syndrome virus, a member of the arterivirus group. Virology.
322 1993;193(1):329-39.
323 5.	Meulenberg JJ, Hulst MM, de Meijer EJ, Moonen PL, den Besten A, de Kluyver EP, et al. Lelystad
324 virus, the causative agent of porcine epidemic abortion and respiratory syndrome (PEARS), is related to
325 LDV and EAV. Virology. 1993;192(1):62-72.
326 6.	Neumann EJ, Kliebenstein JB, Johnson CD, Mabry JW, Bush EJ, Seitzinger AH, et al. Assessment
327 of the economic impact of porcine reproductive and respiratory syndrome on swine production in the
328 United States. J Am Vet Med Assoc. 2005;227(3):385-92.
329 7.	Nauwynck HJ, Duan X, Favoreel HW, Van Oostveldt P, Pensaert MB. Entry of porcine
330 reproductive and respiratory syndrome virus into porcine alveolar macrophages via receptor-mediated
331 endocytosis. J Gen Virol. 1999;80 ( Pt 2):297-305.
332 8.	Calvert JG, Slade DE, Shields SL, Jolie R, Mannan RM, Ankenbauer RG, et al. CD163 expression
333 confers	susceptibility	to	porcine	reproductive	and	respiratory	syndrome	viruses.	J	Virol.
334 2007;81(14):7371-9.
335 9.	Shi C, Liu Y, Ding Y, Zhang Y, Zhang J. PRRSV receptors and their roles in virus infection. Arch
336 Microbiol. 2015;197(4):503-12.
337 10.	Wei X, Li R, Qiao S, Chen XX, Xing G, Zhang G. Porcine Reproductive and Respiratory Syndrome
338 Virus Utilizes Viral Apoptotic Mimicry as an Alternative Pathway To Infect Host Cells. J Virol. 2020;94(17).
339 11.	Kang YL, Oh C, Ahn SH, Choi JC, Choi HY, Lee SW, et al. Inhibition of endocytosis of porcine
340 reproductive and respiratory syndrome virus by rottlerin and its potential prophylactic administration
341 in piglets. Antiviral Res. 2021;195:105191.
342 12.	Shabir N, Khatun A, Nazki S, Kim B, Choi EJ, Sun D, et al. Evaluation of the Cross-Protective
343 Efficacy of a Chimeric Porcine Reproductive and Respiratory Syndrome Virus Constructed Based on Two
344 Field Strains. Viruses. 2016;8(8).
345 13.	Zhu L, Bi J, Zheng L, Zhao Q, Shu X, Guo G, et al. In vitro inhibition of porcine reproductive
346 and respiratory syndrome virus replication by short antisense oligonucleotides with locked nucleic acid
347 modification. BMC Vet Res. 2018;14(1):109.
348 14.	Choi K, Ryu SW, Song S, Choi H, Kang SW, Choi C. Caspase-dependent generation of reactive
349 oxygen species in human astrocytoma cells contributes to resistance to TRAIL-mediated apoptosis. Cell
350 Death Differ. 2010;17(5):833-45.
351 15.	Wernike K, Hoffmann B, Dauber M, Lange E, Schirrmeier H, Beer M. Detection and typing of
352 highly pathogenic porcine reproductive and respiratory syndrome virus by multiplex real-time rt-PCR.
353 PLoS One. 2012;7(6):e38251.
354 16.	Kim WI, Lee DS, Johnson W, Roof M, Cha SH, Yoon KJ. Effect of genotypic and biotypic
355 differences among PRRS viruses on the serologic assessment of pigs for virus infection. Vet Microbiol.
356 2007;123(1-3):1-14.
357 17.	Jayashankar V, Edinger AL. Macropinocytosis confers resistance to therapies targeting cancer
358 anabolism. Nat Commun. 2020;11(1):1121.
359 18.	Falcone S, Cocucci E, Podini P, Kirchhausen T, Clementi E, Meldolesi J. Macropinocytosis:
360 regulated coordination of endocytic and exocytic membrane traffic events. J Cell Sci. 2006;119(Pt
361 22):4758-69.
362 19.	Nakase I, Hirose H, Tanaka G, Tadokoro A, Kobayashi S, Takeuchi T, et al. Cell-surface
363 accumulation of flock house virus-derived peptide leads to efficient internalization via macropinocytosis.
364 Mol Ther. 2009;17(11):1868-76.
365 20.	Commisso C, Flinn RJ, Bar-Sagi D. Determining the macropinocytic index of cells through a
366 quantitative image-based assay. Nat Protoc. 2014;9(1):182-92.
367 21.	Singla B, Lin HP, Ghoshal P, Cherian-Shaw M, Csanyi G. PKCdelta stimulates macropinocytosis
368 via activation of SSH1-cofilin pathway. Cell Signal. 2019;53:111-21.
369 22.	Ghosh M, Song X, Mouneimne G, Sidani M, Lawrence DS, Condeelis JS. Cofilin promotes actin
370 polymerization and defines the direction of cell motility. Science. 2004;304(5671):743-6.
371 23.	Hotulainen P, Paunola E, Vartiainen MK, Lappalainen P. Actin-depolymerizing factor and cofilin-
372 1 play overlapping roles in promoting rapid F-actin depolymerization in mammalian nonmuscle cells.
373 Mol Biol Cell. 2005;16(2):649-64.
374 24.	Po'uha ST, Shum MS, Goebel A, Bernard O, Kavallaris M. LIM-kinase 2, a regulator of actin
375 dynamics, is involved in mitotic spindle integrity and sensitivity to microtubule-destabilizing drugs.
376 Oncogene. 2010;29(4):597-607.
377 25.	Mardilovich K, Baugh M, Crighton D, Kowalczyk D, Gabrielsen M, Munro J, et al. LIM kinase
378 inhibitors disrupt mitotic microtubule organization and impair tumor cell proliferation. Oncotarget.
379 2015;6(36):38469-86.
380 26.	Contreras X, Mzoughi O, Gaston F, Peterlin MB, Bahraoui E. Protein kinase C-delta regulates
381 HIV-1 replication at an early post-entry step in macrophages. Retrovirology. 2012;9:37.
382 27.	Zhou S, Lin Q, Huang C, Luo X, Tian X, Liu C, et al. Rottlerin plays an antiviral role at early and
383 late steps of Zika virus infection. Virol Sin. 2022;37(5):685-94.
384 28.	Gobeil LA, Lodge R, Tremblay MJ. Macropinocytosis-like HIV-1 internalization in macrophages
385 is CCR5 dependent and leads to efficient but delayed degradation in endosomal compartments. J Virol.
386 2013;87(2):735-45.
387 29.	Li M, Zhang D, Li C, Zheng Z, Fu M, Ni F, et al. Characterization of Zika Virus Endocytic Pathways
388 in Human Glioblastoma Cells. Front Microbiol. 2020;11:242.
389 30.	Daecke J, Fackler OT, Dittmar MT, Kräusslich HG. Involvement of clathrin-mediated endocytosis
390 in human immunodeficiency virus type 1 entry. J Virol. 2005;79(3):1581-94.
391 31.	Huang TY, DerMardirossian C, Bokoch GM. Cofilin phosphatases and regulation of actin
392 dynamics. Curr Opin Cell Biol. 2006;18(1):26-31.
393 32.	Bamburg JR. Proteins of the ADF/cofilin family: essential regulators of actin dynamics. Annu
394 Rev Cell Dev Biol. 1999;15:185-230.
395 33.	Prunier C, Prudent R, Kapur R, Sadoul K, Lafanechere L. LIM kinases: cofilin and beyond.
396 Oncotarget. 2017;8(25):41749-63.
397 34.	Yang N, Higuchi O, Ohashi K, Nagata K, Wada A, Kangawa K, et al. Cofilin phosphorylation by
398 LIM-kinase 1 and its role in Rac-mediated actin reorganization. Nature. 1998;393(6687):809-12.
399 35.	Gorovoy M, Niu J, Bernard O, Profirovic J, Minshall R, Neamu R, et al. LIM Kinase 1 Coordinates
400 Microtubule Stability and Actin Polymerization in Human Endothelial Cells*. Journal of Biological
401 Chemistry. 2005;280(28):26533-42.
402 36.	Zhao H, Guo XK, Bi Y, Zhu Y, Feng WH. PKCdelta is required for porcine reproductive and
403 respiratory syndrome virus replication. Virology. 2014;468-470:96-103.
404 37.	Vorster PJ, Guo J, Yoder A, Wang W, Zheng Y, Xu X, et al. LIM kinase 1 modulates cortical actin
405 and CXCR4 cycling and is activated by HIV-1 to initiate viral infection. J Biol Chem. 2011;286(14):12554-
406 64.
407 38.	Yi F, Guo J, Dabbagh D, Spear M, He S, Kehn-Hall K, et al. Discovery of Novel Small-Molecule
408 Inhibitors of LIM Domain Kinase for Inhibiting HIV-1. J Virol. 2017;91(13).
409 39.	Srivastava MC, Singh SW, Tewari JP. Anthelmintic activity of Mallotus philippinensis-kambila
410 powder. Indian J Med Res. 1967;55(7):746-8.
411 40.	Gschwendt M, Muller HJ, Kielbassa K, Zang R, Kittstein W, Rincke G, et al. Rottlerin, a novel
412 protein kinase inhibitor. Biochem Biophys Res Commun. 1994;199(1):93-8.
413 41.	Bain J, Plater L, Elliott M, Shpiro N, Hastie CJ, McLauchlan H, et al. The selectivity of protein
414 kinase inhibitors: a further update. Biochem J. 2007;408(3):297-315.
415 42.	Soltoff SP. Rottlerin Is a Mitochondrial Uncoupler That Decreases Cellular ATP Levels and
416 Indirectly Blocks Protein Kinase C&#x3b4; Tyrosine Phosphorylation *. Journal of Biological Chemistry.
417 2001;276(41):37986-92.
418 43.	Ojha D, Winkler CW, Leung JM, Woods TA, Chen CZ, Nair V, et al. Rottlerin inhibits La Crosse
419 virus-induced encephalitis in mice and blocks release of replicating virus from the Golgi body in neurons.
420 Nat Microbiol. 2021;6(11):1398-409.
421 44.	Song KS, Kim JS, Yun EJ, Kim YR, Seo KS, Park JH, et al. Rottlerin induces autophagy and
422 apoptotic cell death through a PKC-delta-independent pathway in HT1080 human fibrosarcoma cells:
423 the protective role of autophagy in apoptosis. Autophagy. 2008;4(5):650-8.
424 45.	Sugawara T, Nakatsu D, Kii H, Maiya N, Adachi A, Yamamoto A, et al. PKCdelta and epsilon
425 regulate the morphological integrity of the ER-Golgi intermediate compartment (ERGIC) but not the
426 anterograde and retrograde transports via the Golgi apparatus. Biochim Biophys Acta. 2012;1823(4):861-
427 75.

428

###### 429 Fig. 1. Rottlerin inhibits PKCδ-mediated PRRSV cellular entry.

430 (A–C) MARC-145 cells were silenced with siRNA targeting PKCδ, or treated with rottlerin (5
431 μM), or EIPA (50 μM), inoculated with PRRSV FL12 (MOI = 1), and incubated for 48 hours at
432 37 °C in 5% CO2. (A and C) Virus titers in cell supernatants were quantified by qRT-PCR (A) or
433 titration (C). (B) Cells were washed with PBS, lysed, subjected to SDS-PAGE, and transferred
434 onto a nitrocellulose membrane. The blots were incubated with mouse anti-N monoclonal
435 antibodies or mouse anti-β-actin-HRP, followed by goat anti-mouse IgG-HRP. (D) Semi-confluent
436 MARC-145 cells were pretreated with DMSO, rottlerin (5 μM), or EIPA (50 μM) at 37°C for 2
437 hours, inoculated with PRRSV FL12 (MOI = 50), and incubated for 1 hour. Cells were washed
438 and treated with proteinase K to remove extracellular viruses for 45 minutes at 4°C. After fixation
439 and permeabilization, the cells were probed with anti-N monoclonal antibodies, followed by goat
440 anti-mouse IgG and Alexa Fluor 488 at 37 ℃. Nuclei were stained with Hoechst 33342.
441 Statistically significant differences were measured using Kruskal–Wallis nonparametric tests and
442 are indicated by asterisks (*, p < 0.05; **, p < 0.01). Scale bars, 5 μm. PKCδ, protein kinase Cδ;
443 PRRSV, porcine reproductive and respiratory syndrome virus; EIPA, 5-[*N*-ethyl-*N*-isopropyl]
444 amiloride; MOI, a multiplicity of infection; PBS, phosphate-buffered saline; SDS-PAGE, sodium
445 dodecyl sulfate-polyacrylamide gel electrophoresis; N, nucleocapsid; HRP, actin-horseradish
446 peroxidase; qRT-PCR, quantitative reverse transcription–polymerase chain reaction; DMSO,

447

448

dimethyl sulfoxide.

###### 449 Fig. 2. PRRSV enters MARC-145 cells via macropinocytosis and rottlerin blocks the

450 **macropinocytosis by decreasing actin polymerization.**
451 (A-C) Semi-confluent MARC-145 cells were pretreated with DMSO, rottlerin (5 μM), or EIPA
452 (50 μM) for 2 hours at 37 °C. (A) The cells were washed and then inoculated with CF®640R
453 dextran (10,000 MW) for 1 hour. Nuclei were stained with Hoechst 33342. (B-C) DMSO-, rottlerin
454 (5 μM)-, or EIPA (50 μM)-pretreated MARC-145 cells were washed and then inoculated with
455 PRRSV FL12 (MOI= 50) and CF®640R dextran (10,000 MW) for 1 hour. Cells were washed with
456 sodium acetate buffer (pH 5.2) to remove extracellular signals. After fixation and permeabilization,
457 cells were probed with anti-N monoclonal antibodies, followed by goat anti-mouse IgG and Alexa
458 Fluor 488 at 37 ℃. Nuclei were stained with Hoechst 33342. Scale bars, 5 μm. (C) Co-localization
459 of signals between dextran and PRRSV N protein in panel B was measured by Pearson’s
460 coefficient. (D) DMSO-, rottlerin (5 μM)-, or EIPA (50 μM)-pretreated MARC-145 cells were
461 inoculated with PRRSV FL12 (MOI= 50) and incubated with the indicated drug for 1 hour. After
462 fixation, cells were probed with CF®640R phalloidin at 37 ℃. Nuclei were stained with Hoechst
463 33342. Pseudopods resulting from actin rearrangement are marked as white arrows. (E) Cells with
464 clear pseudopods were counted as positive, whereas cells without pseudopods or with small rare
465 pseudopods were negative. Statistically significant differences are indicated by asterisks ( *, *p*-
466 value <0.05; **, *p*-value <0.01). DMSO, dimethyl sulfoxide; EIPA, 5-[*N*-ethyl-*N*-isopropyl]
467 amiloride; PRRSV, porcine reproductive and respiratory syndrome virus; MOI, multiplicity of

468

469

infection; N, nucleocapsid.

###### 470 Fig. 3. Rottlerin reverses PRRSV-induced activation of cofilin and LIMK1.

471 (A and B) cofilin or LIMK1 was knocked down using siRNA targeting each protein. Then, cells
472 were inoculated with PRRSV FL12 (MOI = 1) for 1 hour and vigorously washed with PBS. After
473 2 days of incubation, cell lysates were subjected to SDS-PAGE, transferred onto nitrocellulose
474 membrane, and blotted for mouse anti-cofilin, rabbit anti-LIMK1, or mouse anti-β-actin-HRP,
475 followed by goat anti-rabbit IgG-HRP or goat anti-mouse IgG-HRP. (B) Viral levels in cell
476 supernatants of the cells were quantified by titration. (C-F) Confluent MARC-145 cells were
477 inoculated with PRRSV FL12 (MOI= 1) and incubated with rottlerin (5 μM) for the indicated time
478 period at 37 °C in 5% CO2. After washing with PBS, cell lysates were subjected to SDS-PAGE
479 and transferred onto a nitrocellulose membrane. The membranes were incubated with rabbit anti-
480 cofilin (P-cofilin, Ser3), mouse anti-cofilin (total cofilin), rabbit anti-LIMK1/2 (P-LIMK1,
481 Thr508/505), rabbit anti-LIMK1 (total LIMK1), or mouse anti-β-actin-HRP, followed by goat anti-
482 rabbit IgG-HRP or goat anti-mouse IgG-HRP. (D and F) Densitometry analysis plots of the kinetics
483 of the relative levels of P-LIMK1/total LIMK1 (D) or P-cofilin/total cofilin (F) after being
484 normalized by β-actin. PRRSV, porcine reproductive and respiratory syndrome virus; LIMK1,
485 LIM domain kinase 1; PBS, phosphate-buffered saline; SDS-PAGE, sodium dodecyl sulfate-
486 polyacrylamide gel electrophoresis; HRP, horseradish peroxidase; P-cofilin, phospho-cofilin; P-
487 LIMK1, phospho-LIMK1; siRNA, small interfering RNA; MOI, multiplicity of infection; mpi,
488 minute post-infection.

Unmarked unhighlighted Manuscript	[Click here to access/download;Manuscript;Unmarked unhighted](https://www2.cloud.editorialmanager.com/pone/download.aspx?id=38216008&guid=0df7ffc9-3a43-4f84-a0cc-b16835ecfa09&scheme=1)[<https://www2.cloud.editorialmanager.com/pone/download.aspx?id=38216008&guid=0df7ffc9-3a43-4f84-a0cc-b16835ecfa09&scheme=1> Manuscript.docx](https://www2.cloud.editorialmanager.com/pone/download.aspx?id=38216008&guid=0df7ffc9-3a43-4f84-a0cc-b16835ecfa09&scheme=1)

1 **Rottlerin inhibits macropinocytosis of Porcine Reproductive and**
2 **Respiratory Syndrome Virus through the PKCδ-Cofilin signaling**
3 **pathway**

4

5	Yeonglim Kang^a^, Jong-Chul Choi^a^, Joong-Bok Lee^a^, Seung-Yong Park^a^, and Changin Oh^b*^

6

7 a*Laboratory of Infectious Diseases, College of Veterinary Medicine, Konkuk University, Seoul,*
8 *Republic of Korea*
9 b*Department of Genetics, Yale University School of Medicine, New Haven, Connecticut, United*
10 *States of America*

11

###### 12 *Corresponding author

13 E-mail: [changin.oh@yale.edu](mailto:changin.oh@yale.edu) (CO)

14

15

16

17

18

19	**Abstract**

20 Rottlerin exerts antiviral activity against various enveloped viruses, yet the mechanism by which
21 it inhibits viral replication and the associated signaling pathways remains unclear. Here, we
22 investigated the mechanisms for the antiviral effects of Rottlerin against Porcine Reproductive and
23 Respiratory Syndrome Virus (PRRSV) in vitro. We demonstrate that PRRSV enters host cells via
24 macropinocytosis. Rottlerin, a PKCδ inhibitor, partially inhibits PRRSV entry by decreasing actin
25 polymerization. LIM domain kinase 1 (LIMK1) is essential for PRRSV replication, and PRRSV
26 infection-induced cofilin activation, which was reversed by rottlerin treatment. Our findings
27 suggest that a subset of PRRSV utilizes PKCδ-mediated actin dynamics to enter cells via
28 macropinocytosis, and that rottlerin is a potential antiviral molecule targeting this entry pathway.

29

###### 30 Keywords

31 PKCδ, rottlerin, PRRSV, antivirals, macropinocytosis

32

## 33 Introduction

34 Macropinocytosis is an endocytic process that leads to the internalization of fluids, extracellular
35 particles, and membranes into large vacuoles (1). Activation of actin and microfilaments induces
36 macropinocytosis through the formation of lamellipodia, membrane ruffles, and blebs on the
37 cellular periphery (2). Various viral families, such as human immunodeficiency virus (HIV),
38 vaccinia virus, and adenovirus, and various types of bacteria take advantage of macropinocytosis
39 as a direct means of cellular internalization or an indirect mechanism to assist the penetration of
40 particles that have entered by other forms of endocytosis (2). Thus, blocking macropinocytosis
41 may be a potential strategy for controlling broad-spectrum viral infections.
42 Porcine reproductive and respiratory syndrome virus (PRRSV) is an enveloped positive-sense
43 ssRNA virus belonging to the family Arteriviridae and order Nidovirales (3, 4). PRRSV infection
44 causes reproductive impairments in sows and respiratory diseases in pigs of all ages, resulting in
45 a critical economic impact on the global swine industry (5, 6). It has been reported that PRRSV
46 enters into host cells via low pH-dependent, clathrin-mediated endocytosis, utilizing CD163 as an
47 essential receptor (7-9). A recent paper claims that PRRSV uses macropinocytosis as an alternative
48 entry route (10). However, Additional evidence and detailed mechanisms underlying PRRSV-
49 induced macropinocytosis are necessary to support this novel finding. We recently demonstrated
50 that rottlerin blocks PRRSV endocytosis by inhibiting PRRSV-induced protein kinase C delta
51 (PKCδ) phosphorylation (11), suggesting that inhibition of PKCδ phosphorylation suppresses one
52 or more PRRSV endocytosis routes. Thus, we investigated the antiviral mechanisms of rottlerin
53 against PRRSV, especially how rottlerin blocks PRRSV endocytosis.
54 Here, we demonstrated that PRRSV enters MARC-145 cells via macropinocytosis during infection,
55 which is inhibited by rottlerin treatment, by decreasing actin polymerization. PRRSV
56 macropinocytosis is mediated by the PKCδ-cofilin pathway and LIM domain kinase 1 (LIMK1).
57 The results of the present study provide a possible explanation for the antiviral effect of rottlerin
58 on other viruses and contribute to an understanding of the PRRSV entry mechanism.

59

## 60 Materials and Methods

61 **Viruses**
62 The PRRSV FL12 strain used in this study was rescued from the pCMV-FL12/24 plasmid that was
63 kindly provided by Professor Won-Il Kim (12). Briefly, semi-confluent MARC-145 cells were
64 transfected with pCMV-FL12/24 and the ViaFect reagent (Promega, Madison, WI, USA) in a 1:3
65 ratio. After 72 hours of transfection, cell supernatants were harvested and passaged into MARC-
66 145 cells. Viral titers were measured using quantitative reverse transcription–polymerase chain
67 reaction (qRT-PCR) and titration. Viral stocks were stored at −80 ℃. Viruses were concentrated
68 by centrifugation using a Beckman SW-41 Ti rotor at 160,000 × *g* at 4 ℃ for 4 hours through a
69 discontinuous sucrose gradient consisting of 1 mL of 25%, 35%, 45%, 55%, and 65% sucrose. The
70 35%–45% fraction was dialyzed and titrated for future experiments.

71

72 **Immunofluorescence assay and confocal microscopy**
73 Approximately 2.4 × 10^4^ MARC-145 cells per well were seeded on an eight-chamber slide. On
74 the following day, MARC-145 cells were preincubated with rottlerin (5 μM) or 5-[*N*-ethyl-*N*-
75 isopropyl] amiloride (EIPA) (50 μM) (Tocris, Bristol, UK) at 37 °C for 2 hours or left untreated.
76 For the viral endocytosis assay, cells were infected with PRRSV FL12 at a multiplicity of infection
77 (MOI) of 50 for 1 hour. Cells were washed and treated with proteinase K to remove extracellular
78 viruses for 45 minutes at 4°C. For the dextran uptake assay, cells were inoculated with CF®640R
79 dextran (10,000 MW) (Biotium, Fremont, CA, USA) with or without PRRSV FL12 (MOI = 50),
80 incubated for 1 hour, and washed with 3 M sodium acetate buffer (pH 5.2). The cells were fixed
81 with 4% paraformaldehyde for 15 minutes and permeabilized with Tris-buffered saline (TBS)
82 containing 0.2% Triton X-100 at room temperature (RT) for 10 minutes. The cells were then
83 probed as previously described (13) with minor modifications. CF®640R phalloidin (Biotium,
84 Fremont, CA, USA) was used to label the F-actin. An anti-PRRSV nucleocapsid (N) monoclonal
85 antibody (1:200) (Median Diagnostics, Chuncheon, Korea) was used to label the viral particles.
86 The cells were scanned using an EVOS FL Cell Imaging System (Thermo Fisher Scientific,
87 Waltham, MA, USA) or an LSM 900 with Airyscan 2 (Zeiss, Oberkochen, Germany). The images
88 were processed, and the corrected total cell fluorescence was calculated using ImageJ software
89 (NIH, Bethesda, MD, USA).

90

91 **RNA interference**
92 To silence PKCδ, LIMK1, and cofilin expression, MARC-145 cells were transfected with small
93 interfering RNAs (siRNAs) targeting PKCδ, LIMK1, and cofilin, respectively (14) using
94 Lipofectamine 3000 (Thermo Fisher Scientific) according to the manufacturer’s instructions, and
95 the cells were incubated at 37 °C for 24 hours.

96

97 **Western blot**
98 To test the effects of rottlerin on the phosphorylation of LIMK1 and cofilin, confluent MARC-145
99 cells were treated with rottlerin (5 μM) or EIPA (50 μM), respectively, for 2 hours at 37 ℃. The
100 cells were inoculated with PRRSV FL12 (MOI = 1) and incubated for the indicated times. Cells
101 were lysed using mammalian protein extraction reagent (Thermo Fisher Scientific) with a Halt
102 protease phosphatase inhibitor cocktail (Thermo Fisher Scientific). Cell lysates were resolved
103 using sodium dodecyl sulfate-polyacrylamide gel electrophoresis (Bolt 4%–12% Bis-Tris gel;
104 Invitrogen, Waltham, MA, USA) and transferred onto nitrocellulose membranes (Komabiotech,
105 Seoul, Korea). The membranes were blocked with 5% skim milk in TBS with Tween 20 (TBS-T)
106 for 30 minutes at RT and probed with rabbit anti-cofilin (P-cofilin, Ser3) monoclonal antibody
107 (1:1,000) (Cell Signaling Technology, Danvers, MA, USA), mouse anti-cofilin monoclonal
108 antibody–horseradish peroxidase (HRP) (1:1,000) (Santa Cruz Biotechnology, Dallas, TX, USA),
109 rabbit anti-LIMK1/2 (P-LIMK1, Thr508/505) polyclonal antibody (1:1,000) (BioVision, Waltham,
110 MA, USA), rabbit anti-LIMK1 polyclonal antibody (1:1,000) (Cell Signaling Technology), or
111 mouse anti-β-actin monoclonal antibody-HRP (1:50,000) (Abcam, Cambridge, UK) overnight at
112 4 ℃. After five washes with TBS-T, the membranes were incubated with HRP-conjugated anti-
113 rabbit IgG (Komabiotech) (1:5,000) for 1 hour at RT. Proteins were visualized using Clarity
114 Western ECL Substrate (Bio-Rad, Hercules, CA, USA) and detected using FUSION Solo S (Vilber,
115 Collégien, France). The density of the protein bands was measured using ImageJ software (NIH)
116 after subtracting the density of the β-actin bands.

117

118 **qRT-PCR and virus titration**
119 Viral RNA was extracted from the supernatants of PRRSV-infected cells using an RNeasy Mini
120 Kit (QIAGEN, Hilden, Germany), according to the manufacturer's recommendations. Viral RNA
121 levels were quantified using the RNA UltraSense One-Step Quantitative RT-PCR System
122 (Invitrogen) with the LightCycler 96 System (Roche, Basel, Switzerland), as previously described
123 (15). The measured cycle threshold (CT) values were converted to RNA copy numbers and tissue
124 culture infective dose 50% (TCID50) equivalent/mL using a standard curve generated from CT
125 values plotted against the corresponding TCID50/mL. The viral titer (TCID50/mL) of the collected
126 samples was measured using the Reed–Muench method, as previously described (16).

127

128 **Statistical analyses**
129 All data were analyzed using IBM SPSS Statistics 25 (IBM Corp., Armonk, NY, USA) and
130 GraphPad Prism 6 (GraphPad Software, San Diego, CA, USA) using a nonparametric model
131 (Kruskal–Wallis followed by Mann–Whitney). All data are presented as the mean ± standard
132 deviation, and *, *p*-value <0.0332; **, *p*-value <0.0021; ***, *p*-value <0.0002; ****, *p*-value
133 <0.0001 were considered significant.

134

135

136

137

138

139	**Results**

140 **Rottlerin inhibits PRRSV endocytosis in MARC-145 cells**
141 We previously reported that rottlerin inhibits PRRSV endocytosis(11), but the detailed mechanism
142 of its antiviral activity and involvement of other PKCδ related cellular molecules is unknown. To
143 investigate the role of the PKCδ and elucidate the antiviral mechanism of rottlerin during PRRSV
144 infection, we examined PRRSV replication in the presence of rottlerin in MARC-145 cells. The
145 cells were pre-treated with rottlerin (5 μM) or EIPA (50 μM), or subjected to PKCδ silencing,
146 followed by infection with PRRSV FL12 (MOI= 1). Subsequently, viral replication was evaluated
147 using three distinct parameters at 48 hours post-infection (hpi): PRRSV RNA synthesis, PRRSV
148 nucleocapsid (N) protein expression, and infectious virus production. Consistent with our previous
149 report, both rottlerin and siRNA treatment significantly inhibited viral RNA synthesis (Fig 1A),
150 expression of the N protein (Fig 1B), and production of infectious progeny viruses (Fig 1C).
151 To further determine whether rottlerin blocks PRRSV endocytosis, we used immunofluorescence
152 to observe the localization of PRRSV particles in infected MARC-145 cells after treatment with
153 rottlerin or RIPA. The cells were washed with sodium acetate buffer (pH 5.2) to eliminate the
154 extracellular viruses. As expected, confocal microscopy images of dimethyl sulfoxide (DMSO)-
155 treated cells showed localization of PRRSV to the cytoplasm with a puncta shape (Fig 1D, left),
156 whereas rottlerin-treated cells showed the absence of a signal in the cytoplasm (Fig 1D, middle),
157 similar to EIPA treated cells (Fig 1D, right). This indicates that rottlerin treatment impaired PRRSV
158 endocytosis. These results are consistent with our previous findings showing that rottlerin
159 treatment suppressed PRRSV replication in cells and pigs by inhibiting PRRSV endocytosis (11).
160 **Rottlerin inhibited macropinocytosis of PRRSV by decreasing actin**
161 **polymerization**
162 Due to the inhibition of PRRSV replication and endocytosis by EIPA treatment (Fig 1), a selective
163 macropinocytosis	inhibitor	(17),	we	investigated	whether	PRRSV	entry	occurs	via
164 macropinocytosis and if rottlerin inhibits PRRSV macropinocytosis. We co-incubated MARC-145
165 cells for an hour with fluorescence-labeled CF®640R dextran (10,000 MW), which is known to
166 enter cells via macropinocytosis (18, 19) (Fig 2A). In DMSO-treated cells, dextran signals were
167 observed in the cytoplasm, suggesting macropinocytosis of dextran (Fig 2A, top). In contrast,
168 rottlerin or EIPA-treated cells showed reduced dextran signals around the nucleus (Fig 2A, middle
169 and bottom), indicating that rottlerin also inhibits macropinocytosis.
170 Next, PRRSV and dextran were co-inoculated into DMSO, rottlerin, or EIPA-treated cells. In
171 DMSO-treated cells, signals for both dextran and PRRSV were observed around the nucleus with
172 some colocalization (Fig 2B, top row). This suggests that during the endocytosis of the virus and
173 dextran, some of them enter together through macropinocytosis. In contrast, either rottlerin or EIPA
174 treatment significantly decreased the number of both signals in cells with significantly less co-
175 localization between them (Fig 2C, Pearson’s coefficient: DMSO, 0.43 vs. rottlerin, 0.07). These
176 results suggest that rottlerin inhibited PRRSV macropinocytosis in MARC-145 cells.
177 Among the several characteristics of macropinocytosis, actin polymerization is a key factor for
178 macropinocytosis-specific uptake (10, 20). Therefore, we tested whether rottlerin reduced actin
179 polymerization during PRRSV macropinocytosis similar to EIPA. Actin dynamics were observed
180 using fluorescently labeled phalloidin, a specific ligand of F-actin. DMSO-treated cells after
181 PRRSV infection showed multiple pseudopods projecting from the plasma membrane into the
182 outer space (white arrow) (Fig 2D, top row). Interestingly, rottlerin or EIPA treatment caused the
183 disappearance of these projections (Fig 2D, middle and bottom rows). Quantitative analysis
184 showed that cells with proper projections were significantly decreased when treated with rottlerin
185 (*p*-value < 0.05) and EIPA (*p*-value < 0.01) compared with the DMSO-treated cells (Fig 2E),
186 confirming that rottlerin inhibited PRRSV macropinocytosis by reducing actin polymerization.

187

188 **Rottlerin inhibits PRRSV endocytosis through the PKCδ-cofilin**
189 **signaling pathway**
190 PKCδ regulates actin dynamics during macropinocytosis via downstream effectors such as cofilin,
191 slingshot phosphatase homolog 1, and LIMK1(21). Cofilin regulates the dynamics of the actin
192 cytoskeleton by binding to an actin filament(22, 23). LIMK1 directly phosphorylates cofilin at Ser-
193 3, which inactivates it (21, 24, 25). To evaluate the involvement of cofilin and LIMK1 in PRRSV
194 macropinocytosis, cofilin- or LIMK1-silenced MARC-145 cells were used to measure PRRSV
195 replication. Transfection of siRNAs targeting cofilin or LIMK1 effectively knocked down the
196 expression of each protein in the cells (Fig 3A). Interestingly silencing LIMK1 resulted in
197 decreased expression of cofilin.
198 Next, we investigated the effect of cofilin or LIMK1 silencing on PRRSV replication by measuring
199 viral titer and expression of the N protein in siRNA-treated cells after PRRSV infection. The N
200 protein blot showed that silencing LIMK1 significantly inhibited N protein synthesis with the
201 relative intensity of the band being 0.36 compared to treatment with siNC (Fig 3A, Relative N
202 protein intensity:1.00). Silencing Cofilin resulted in an intensity of 0.84 for the N protein band,
203 but this effect was not as significant as that observed with LIMK1 silencing. Consistent with the
204 western blot results, the silencing of LIMK1 notably reduced the viral titer by 11-fold compared
205 to siNC-treated cells (Fig 3B). Cofilin silencing also significantly decreased the viral titer but less
206 than that of siLIMK1. These results suggest that both LIMK1 and cofilin serves as a host
207 dependency factor essential for PRRSV infection.
208 To further investigate how macropinocytosis is regulated by rottlerin following PRRSV infection,
209 we monitored the activity of cofilin or LIMK1 in PRRSV-infected cells. Since the activities of
210 LIMK1 and cofilin are regulated by phosphorylation, we examined the levels of phosphorylated
211 LIMK1 and cofilin (p-LIMK1 and p-cofilin) as well as total LIMK1 and cofilin using immunoblots.
212 As shown in Fig 3C and D, the levels of p-LIMK1 after PRRSV infection with DMSO transiently
213 increased immediately after PRRSV infection (15 mpi), slightly dephosphorylated (30 mpi), and
214 then re-phosphorylated at a later time point (120 mpi). In contrast, rottlerin treatment rapidly
215 attenuated the virus-induced p-LIMK1, and the p-LIMK1 levels in rottlerin-treated cells were
216 returned similar to Mock-treated cells at 30, and 120 mpi (Fig 3C and D). Similar levels of total
217 LIMK1 after DMSO or rottlerin treatment suggest that the changes in p- LIMK1 were not due to
218 a change in total LIMK1. These results suggest that PRRSV infection mediates transient activation
219 of LIMK1 at an early stage of infection through PKCδ, which was rapidly attenuated by rottlerin.
220 PRRSV infection gradually decreased p-cofilin levels in DMSO-treated cells, and a low level of
221 p-cofilin was maintained up to 120 mpi (Fig 3E and F). In contrast, rottlerin treatment led to
222 increase p- cofilin. The levels of p-cofilin in rottlerin-treated cells began to increase at 15 mpi and
223 continued to rise until 120 mpi (Fig 3E and F). Quantitative analysis of the blot bands showed that
224 rottlerin treatment significantly increased p-cofilin levels at 90 and 120 mpi compared with DMSO
225 treatment (Fig 3F). These results demonstrate that PRRSV infection decreases P-cofilin levels,
226 whereas rottlerin treatment reverses this effect. Overall, the findings suggest that rottlerin regulates
227 LIMK1 and cofilin signaling pathways, which may contribute to the mechanism by which rottlerin
228 inhibits PRRSV-induced macropinocytosis**.**

229	**Discussion**

230 In this study, our microscopic and biochemical analyses demonstrated that rottlerin treatment
231 inhibited PRRSV replication by decreasing viral endocytosis in vitro, which resulted in the
232 suppression of viral RNA synthesis, N protein expression, and infectious virus production (Fig 1).
233 We also showed that PRRSV entered MARC-145 cells via macropinocytosis, and rottlerin blocked
234 it by decreasing actin polymerization (Fig 2). LIMK1 acts as a host dependency factor crucial for
235 PRRSV infection. Rottlerin reversed PRRSV-induced activation of cofilin (Fig 3). These findings
236 provide a possible explanation for one of the antiviral mechanisms of rottlerin at the early stages
237 of virus infection, i.e., rottlerin blocks macropinocytosis of viruses by decreasing actin
238 polymerization via the PKCδ-cofilin signaling pathway. In the case of HIV1 and Zika virus,
239 rottlerin inhibits an early stage of their replication (26, 27) and they are known to enter cells via
240 actin-related entry pathways, indicating that rottlerin could inhibit their entry through a similar
241 mechanism (28-30).
242 Our results demonstrated that macropinocytosis serves as an alternative route for PRRSV to
243 infect host cells. Our findings match with a recent study that PRRSV infects host cells through
244 macropinocytosis, involving T-cell immunoglobulin and mucin domain proteins as well as CD163
245 (10). We further hypothesize that PRRSV possibly enters cells via at least two distinct pathways:
246 clathrin-mediated endocytosis and macropinocytosis. Few in vitro assay results support the
247 hypothesis that a single treatment with chlorpromazine, a clathrin lattice polymerization inhibitor,
248 or rottlerin partially blocked PRRSV replication (Fig 1C), but the combination of both inhibitors
249 almost completely inhibited PRRSV replication (10). Understanding the potential PRRSV entry
250 routes is crucial for the clinical application of antiviral drugs. To achieve an effective anti-PRRSV
251 treatment, a combination of inhibitors targeting both entry routes, rather than a single treatment
252 with either route inhibitor, is necessary.
253 Our results provide insights into how PRRSV induces macropinocytosis through the PKCδ-
254 LIMK1-cofilin signaling pathway to infect host cells. Although macropinocytosis signaling
255 mechanisms have been extensively studied over the last decade (2, 21), the PRRSV-mediated
256 signaling pathway of macropinocytosis remains unclear. Generally, to induce macropinocytosis by
257 cofilin and LIMK1, the binding of the active form of cofilin (dephosphorylated at Ser3)
258 depolymerizes actin by severing the actin filaments, which finally induces lamellipodium
259 formation and macropinocytosis(31). However, filament severing also results in actin
260 polymerization, depending on the relative amount of actin monomers and free barb ends (32). LIM
261 kinases directly phosphorylate cofilin, which allows the precise regulation of actin dynamics (33).
262 LIMK1 is known to not only phosphorylate cofilin but also directly bind F-actin to induce actin
263 depolymerization and membrane ruffling (34). In the case of PRRSV, we propose that viral
264 infection activates cofilin to increase lamellipodium formation for macropinocytosis, which
265 increases viral uptake from the extracellular space (21). Rottlerin treatment inhibits this activation
266 by blocking upstream PKCδ(11). Our microscopic data is in line with this model (Fig 2).
267 Our results regarding LIMK1 phosphorylation cannot be fully explained by the standard cofilin-
268 LIMK1 pathway. In our study, PRRSV infection induced LIMK1 phosphorylation (Fig 3C and D),
269 which is contrary to what would be expected based on our microscopic and cofilin phosphorylation
270 results. Typically, phosphorylated LIMK1 adds a phosphate group to cofilin, thereby inactivating
271 cofilin and stabilizing actin filaments. Considering all our results, LIMK1 may play an additional
272 role in this virus entry process. It is possible that LIMK1 not only phosphorylates cofilin but also
273 precisely regulates this pathway. Previous research shows LIMK1 is involved in the microtubule
274 destabilization(35), suggesting LIMK1 may directly coordinate microtubules and actin
275 cytoskeleton. Another research also supports this notion, indicating that pharmacological
276 inhibition of LIMK1 does not stimulate macropinocytosis(21). Even if LIMK1 did not follow the
277 standard pathway, it appears that LIMK1 is a key regulator employed by PRRSV to control
278 macropinocytosis. These results are consistent with previous studies showing that PRRSV
279 replication requires PKCδ (36). HIV binding to CD4+ T cells initiates transient activation of LIMK
280 and actin polymerization (37). Small-molecule inhibitors of LIM kinases have been reported to
281 inhibit HIV1 (38). Further research is needed to study the mechanisms underlying virus-induced
282 macropinocytosis.
283 Rottlerin is a secondary metabolite isolated from *Mallotus philippinensis*, which has been
284 traditionally used to treat tapeworm, scabies, and herpetic ringworm infections in India for several
285 centuries (39). Rottlerin is a selective inhibitor of PKCδ (40, 41), but recent studies have reported
286 that rottlerin inhibits multiple kinases (42-44). In our study, PKCδ inhibition of rottlerin is highly
287 likely to be a major blocking mechanism of PRRSV macropinocytosis, rather than other
288 mechanisms, because silencing of PKCδ decreased PRRSV replication (Fig 1), and we have
289 previously shown that rottlerin treatment dephosphorylates PKCδ induced by PRRSV infection
290 (11). Interestingly, several other studies have demonstrated the PKCδ-independent antiviral effects
291 of rottlerin (43, 44). Ojha et al. recently showed that rottlerin inhibits La Crosse virus trafficking
292 from the Golgi apparatus to the trans-Golgi vesicles, which may be related to decreased cellular
293 ATP levels induced by rottlerin (43, 45). These results suggest that rottlerin can block various steps
294 of viral replication, thus strengthening its antiviral potency. Thus, an intensive mechanistic study
295 of the antiviral effects of rottlerin against various viruses is required.
296 In conclusion, the current study demonstrates that PRRSV utilizes macropinocytosis to infect host
297 cells. Rottlerin blocks macropinocytosis of PRRSV related to PKCδ-cofilin signaling pathways by
298 reducing actin polymerization. Our results suggest that rottlerin is a potential antiviral drug
299 targeting macropinocytosis and its associated signaling molecules.

300

301

302	**Acknowledgment**

303 We are deeply grateful to the KU Research Center for Zoonosis for providing advice on the design
304 and progress of this study.

305

306	**References**

307

308

309

310

311

312

313

314

315

316

317

318

319

320

321

322

323

324

325

326

327

328

329

330

331

332

333

334

335

336

337

338

339

340

341

342

343

344

345

346

347

348

349

350

351

352

353

354

355

356

1. Zeineddine R, Yerbury JJ. The role of macropinocytosis in the propagation of protein aggregation associated with neurodegenerative diseases. Front Physiol. 2015;6:277.
2. Mercer J, Helenius A. Virus entry by macropinocytosis. Nat Cell Biol. 2009;11(5):510-20.
3. Cavanagh D. Nidovirales: a new order comprising Coronaviridae and Arteriviridae. Arch Virol. 1997;142(3):629-33.
4. Conzelmann KK, Visser N, Van Woensel P, Thiel HJ. Molecular characterization of porcine reproductive and respiratory syndrome virus, a member of the arterivirus group. Virology. 1993;193(1):329-39.
5. Meulenberg JJ, Hulst MM, de Meijer EJ, Moonen PL, den Besten A, de Kluyver EP, et al. Lelystad virus, the causative agent of porcine epidemic abortion and respiratory syndrome (PEARS), is related to LDV and EAV. Virology. 1993;192(1):62-72.
6. Neumann EJ, Kliebenstein JB, Johnson CD, Mabry JW, Bush EJ, Seitzinger AH, et al. Assessment of the economic impact of porcine reproductive and respiratory syndrome on swine production in the United States. J Am Vet Med Assoc. 2005;227(3):385-92.
7. Nauwynck HJ, Duan X, Favoreel HW, Van Oostveldt P, Pensaert MB. Entry of porcine reproductive and respiratory syndrome virus into porcine alveolar macrophages via receptor-mediated endocytosis. J Gen Virol. 1999;80 ( Pt 2):297-305.
8. Calvert JG, Slade DE, Shields SL, Jolie R, Mannan RM, Ankenbauer RG, et al. CD163 expression confers susceptibility to porcine reproductive and respiratory syndrome viruses. J Virol. 2007;81(14):7371-9.
9. Shi C, Liu Y, Ding Y, Zhang Y, Zhang J. PRRSV receptors and their roles in virus infection. Arch Microbiol. 2015;197(4):503-12.
10. Wei X, Li R, Qiao S, Chen XX, Xing G, Zhang G. Porcine Reproductive and Respiratory Syndrome Virus Utilizes Viral Apoptotic Mimicry as an Alternative Pathway To Infect Host Cells. J Virol. 2020;94(17).
11. Kang YL, Oh C, Ahn SH, Choi JC, Choi HY, Lee SW, et al. Inhibition of endocytosis of porcine reproductive and respiratory syndrome virus by rottlerin and its potential prophylactic administration in piglets. Antiviral Res. 2021;195:105191.
12. Shabir N, Khatun A, Nazki S, Kim B, Choi EJ, Sun D, et al. Evaluation of the Cross-Protective Efficacy of a Chimeric Porcine Reproductive and Respiratory Syndrome Virus Constructed Based on Two Field Strains. Viruses. 2016;8(8).
13. Zhu L, Bi J, Zheng L, Zhao Q, Shu X, Guo G, et al. In vitro inhibition of porcine reproductive and respiratory syndrome virus replication by short antisense oligonucleotides with locked nucleic acid modification. BMC Vet Res. 2018;14(1):109.
14. Choi K, Ryu SW, Song S, Choi H, Kang SW, Choi C. Caspase-dependent generation of reactive oxygen species in human astrocytoma cells contributes to resistance to TRAIL-mediated apoptosis. Cell Death Differ. 2010;17(5):833-45.
15. Wernike K, Hoffmann B, Dauber M, Lange E, Schirrmeier H, Beer M. Detection and typing of highly pathogenic porcine reproductive and respiratory syndrome virus by multiplex real-time rt-PCR. PLoS One. 2012;7(6):e38251.
16. Kim WI, Lee DS, Johnson W, Roof M, Cha SH, Yoon KJ. Effect of genotypic and biotypic differences among PRRS viruses on the serologic assessment of pigs for virus infection. Vet Microbiol. 2007;123(1-3):1-14.
17. Jayashankar V, Edinger AL. Macropinocytosis confers resistance to therapies targeting cancer anabolism. Nat Commun. 2020;11(1):1121.
18. Falcone S, Cocucci E, Podini P, Kirchhausen T, Clementi E, Meldolesi J. Macropinocytosis: regulated coordination of endocytic and exocytic membrane traffic events. J Cell Sci. 2006;119(Pt 22):4758-69.
19. Nakase I, Hirose H, Tanaka G, Tadokoro A, Kobayashi S, Takeuchi T, et al. Cell-surface accumulation of flock house virus-derived peptide leads to efficient internalization via macropinocytosis. Mol Ther. 2009;17(11):1868- 76.
20. Commisso C, Flinn RJ, Bar-Sagi D. Determining the macropinocytic index of cells through a quantitative image-based assay. Nat Protoc. 2014;9(1):182-92.
21. Singla B, Lin HP, Ghoshal P, Cherian-Shaw M, Csanyi G. PKCdelta stimulates macropinocytosis via activation of SSH1-cofilin pathway. Cell Signal. 2019;53:111-21.

357

358

359

360

361

362

363

364

365

366

367

368

369

370

371

372

373

374

375

376

377

378

379

380

381

382

383

384

385

386

387

388

389

390

391

392

393

394

395

396

397

398

399

400

401

402

403

404

405

406

407

408

409

410

411

22. Ghosh M, Song X, Mouneimne G, Sidani M, Lawrence DS, Condeelis JS. Cofilin promotes actin polymerization and defines the direction of cell motility. Science. 2004;304(5671):743-6.
23. Hotulainen P, Paunola E, Vartiainen MK, Lappalainen P. Actin-depolymerizing factor and cofilin-1 play overlapping roles in promoting rapid F-actin depolymerization in mammalian nonmuscle cells. Mol Biol Cell. 2005;16(2):649-64.
24. Po'uha ST, Shum MS, Goebel A, Bernard O, Kavallaris M. LIM-kinase 2, a regulator of actin dynamics, is involved in mitotic spindle integrity and sensitivity to microtubule-destabilizing drugs. Oncogene. 2010;29(4):597- 607.
25. Mardilovich K, Baugh M, Crighton D, Kowalczyk D, Gabrielsen M, Munro J, et al. LIM kinase inhibitors disrupt mitotic microtubule organization and impair tumor cell proliferation. Oncotarget. 2015;6(36):38469-86.
26. Contreras X, Mzoughi O, Gaston F, Peterlin MB, Bahraoui E. Protein kinase C-delta regulates HIV-1 replication at an early post-entry step in macrophages. Retrovirology. 2012;9:37.
27. Zhou S, Lin Q, Huang C, Luo X, Tian X, Liu C, et al. Rottlerin plays an antiviral role at early and late steps of Zika virus infection. Virol Sin. 2022;37(5):685-94.
28. Gobeil LA, Lodge R, Tremblay MJ. Macropinocytosis-like HIV-1 internalization in macrophages is CCR5 dependent and leads to efficient but delayed degradation in endosomal compartments. J Virol. 2013;87(2):735-45.
29. Li M, Zhang D, Li C, Zheng Z, Fu M, Ni F, et al. Characterization of Zika Virus Endocytic Pathways in Human Glioblastoma Cells. Front Microbiol. 2020;11:242.
30. Daecke J, Fackler OT, Dittmar MT, Kräusslich HG. Involvement of clathrin-mediated endocytosis in human immunodeficiency virus type 1 entry. J Virol. 2005;79(3):1581-94.
31. Huang TY, DerMardirossian C, Bokoch GM. Cofilin phosphatases and regulation of actin dynamics. Curr Opin Cell Biol. 2006;18(1):26-31.
32. Bamburg JR. Proteins of the ADF/cofilin family: essential regulators of actin dynamics. Annu Rev Cell Dev Biol. 1999;15:185-230.
33. Prunier C, Prudent R, Kapur R, Sadoul K, Lafanechere L. LIM kinases: cofilin and beyond. Oncotarget. 2017;8(25):41749-63.
34. Yang N, Higuchi O, Ohashi K, Nagata K, Wada A, Kangawa K, et al. Cofilin phosphorylation by LIM- kinase 1 and its role in Rac-mediated actin reorganization. Nature. 1998;393(6687):809-12.
35. Gorovoy M, Niu J, Bernard O, Profirovic J, Minshall R, Neamu R, et al. LIM Kinase 1 Coordinates Microtubule Stability and Actin Polymerization in Human Endothelial Cells*. Journal of Biological Chemistry. 2005;280(28):26533-42.
36. Zhao H, Guo XK, Bi Y, Zhu Y, Feng WH. PKCdelta is required for porcine reproductive and respiratory syndrome virus replication. Virology. 2014;468-470:96-103.
37. Vorster PJ, Guo J, Yoder A, Wang W, Zheng Y, Xu X, et al. LIM kinase 1 modulates cortical actin and CXCR4 cycling and is activated by HIV-1 to initiate viral infection. J Biol Chem. 2011;286(14):12554-64.
38. Yi F, Guo J, Dabbagh D, Spear M, He S, Kehn-Hall K, et al. Discovery of Novel Small-Molecule Inhibitors of LIM Domain Kinase for Inhibiting HIV-1. J Virol. 2017;91(13).
39. Srivastava MC, Singh SW, Tewari JP. Anthelmintic activity of Mallotus philippinensis-kambila powder. Indian J Med Res. 1967;55(7):746-8.
40. Gschwendt M, Muller HJ, Kielbassa K, Zang R, Kittstein W, Rincke G, et al. Rottlerin, a novel protein kinase inhibitor. Biochem Biophys Res Commun. 1994;199(1):93-8.
41. Bain J, Plater L, Elliott M, Shpiro N, Hastie CJ, McLauchlan H, et al. The selectivity of protein kinase inhibitors: a further update. Biochem J. 2007;408(3):297-315.
42. Soltoff SP. Rottlerin Is a Mitochondrial Uncoupler That Decreases Cellular ATP Levels and Indirectly Blocks Protein Kinase C&#x3b4; Tyrosine Phosphorylation *. Journal of Biological Chemistry. 2001;276(41):37986- 92.
43. Ojha D, Winkler CW, Leung JM, Woods TA, Chen CZ, Nair V, et al. Rottlerin inhibits La Crosse virus- induced encephalitis in mice and blocks release of replicating virus from the Golgi body in neurons. Nat Microbiol. 2021;6(11):1398-409.
44. Song KS, Kim JS, Yun EJ, Kim YR, Seo KS, Park JH, et al. Rottlerin induces autophagy and apoptotic cell death through a PKC-delta-independent pathway in HT1080 human fibrosarcoma cells: the protective role of autophagy in apoptosis. Autophagy. 2008;4(5):650-8.
45. Sugawara T, Nakatsu D, Kii H, Maiya N, Adachi A, Yamamoto A, et al. PKCdelta and epsilon regulate the morphological integrity of the ER-Golgi intermediate compartment (ERGIC) but not the anterograde and retrograde transports via the Golgi apparatus. Biochim Biophys Acta. 2012;1823(4):861-75.

###### 412 Fig. 1. Rottlerin inhibits PKCδ-mediated PRRSV cellular entry.

413 (A–C) MARC-145 cells were silenced with siRNA targeting PKCδ, or treated with rottlerin (5
414 μM), or EIPA (50 μM), inoculated with PRRSV FL12 (MOI = 1), and incubated for 48 hours at
415 37 °C in 5% CO2. (A and C) Virus titers in cell supernatants were quantified by qRT-PCR (A) or
416 titration (C). (B) Cells were washed with PBS, lysed, subjected to SDS-PAGE, and transferred
417 onto a nitrocellulose membrane. The blots were incubated with mouse anti-N monoclonal
418 antibodies or mouse anti-β-actin-HRP, followed by goat anti-mouse IgG-HRP. (D) Semi-confluent
419 MARC-145 cells were pretreated with DMSO, rottlerin (5 μM), or EIPA (50 μM) at 37°C for 2
420 hours, inoculated with PRRSV FL12 (MOI = 50), and incubated for 1 hour. Cells were washed
421 and treated with proteinase K to remove extracellular viruses for 45 minutes at 4°C. After fixation
422 and permeabilization, the cells were probed with anti-N monoclonal antibodies, followed by goat
423 anti-mouse IgG and Alexa Fluor 488 at 37 ℃. Nuclei were stained with Hoechst 33342.
424 Statistically significant differences were measured using Kruskal–Wallis nonparametric tests and
425 are indicated by asterisks (*, p < 0.05; **, p < 0.01). Scale bars, 5 μm. PKCδ, protein kinase Cδ;
426 PRRSV, porcine reproductive and respiratory syndrome virus; EIPA, 5-[*N*-ethyl-*N*-isopropyl]
427 amiloride; MOI, a multiplicity of infection; PBS, phosphate-buffered saline; SDS-PAGE, sodium
428 dodecyl sulfate-polyacrylamide gel electrophoresis; N, nucleocapsid; HRP, actin-horseradish
429 peroxidase; qRT-PCR, quantitative reverse transcription–polymerase chain reaction; DMSO,

430

431

dimethyl sulfoxide.

###### 432 Fig. 2. PRRSV enters MARC-145 cells via macropinocytosis and rottlerin blocks the

433 **macropinocytosis by decreasing actin polymerization.**
434 (A-C) Semi-confluent MARC-145 cells were pretreated with DMSO, rottlerin (5 μM), or EIPA
435 (50 μM) for 2 hours at 37 °C. (A) The cells were washed and then inoculated with CF®640R
436 dextran (10,000 MW) for 1 hour. Nuclei were stained with Hoechst 33342. (B-C) DMSO-, rottlerin
437 (5 μM)-, or EIPA (50 μM)-pretreated MARC-145 cells were washed and then inoculated with
438 PRRSV FL12 (MOI= 50) and CF®640R dextran (10,000 MW) for 1 hour. Cells were washed with
439 sodium acetate buffer (pH 5.2) to remove extracellular signals. After fixation and permeabilization,
440 cells were probed with anti-N monoclonal antibodies, followed by goat anti-mouse IgG and Alexa
441 Fluor 488 at 37 ℃. Nuclei were stained with Hoechst 33342. Scale bars, 5 μm. (C) Co-localization
442 of signals between dextran and PRRSV N protein in panel B was measured by Pearson’s
443 coefficient. (D) DMSO-, rottlerin (5 μM)-, or EIPA (50 μM)-pretreated MARC-145 cells were
444 inoculated with PRRSV FL12 (MOI= 50) and incubated with the indicated drug for 1 hour. After
445 fixation, cells were probed with CF®640R phalloidin at 37 ℃. Nuclei were stained with Hoechst
446 33342. Pseudopods resulting from actin rearrangement are marked as white arrows. (E) Cells with
447 clear pseudopods were counted as positive, whereas cells without pseudopods or with small rare
448 pseudopods were negative. Statistically significant differences are indicated by asterisks ( *, *p*-
449 value <0.05; **, *p*-value <0.01). DMSO, dimethyl sulfoxide; EIPA, 5-[*N*-ethyl-*N*-isopropyl]
450 amiloride; PRRSV, porcine reproductive and respiratory syndrome virus; MOI, multiplicity of

451

452

infection; N, nucleocapsid.

###### 453 Fig. 3. Rottlerin reverses PRRSV-induced activation of cofilin and LIMK1.

454 (A and B) cofilin or LIMK1 was knocked down using siRNA targeting each protein. Then, cells
455 were inoculated with PRRSV FL12 (MOI = 1) for 1 hour and vigorously washed with PBS. After
456 2 days of incubation, cell lysates were subjected to SDS-PAGE, transferred onto nitrocellulose
457 membrane, and blotted for mouse anti-cofilin, rabbit anti-LIMK1, or mouse anti-β-actin-HRP,
458 followed by goat anti-rabbit IgG-HRP or goat anti-mouse IgG-HRP. (B) Viral levels in cell
459 supernatants of the cells were quantified by titration. (C-F) Confluent MARC-145 cells were
460 inoculated with PRRSV FL12 (MOI= 1) and incubated with rottlerin (5 μM) for the indicated time
461 period at 37 °C in 5% CO2. After washing with PBS, cell lysates were subjected to SDS-PAGE
462 and transferred onto a nitrocellulose membrane. The membranes were incubated with rabbit anti-
463 cofilin (P-cofilin, Ser3), mouse anti-cofilin (total cofilin), rabbit anti-LIMK1/2 (P-LIMK1,
464 Thr508/505), rabbit anti-LIMK1 (total LIMK1), or mouse anti-β-actin-HRP, followed by goat anti-
465 rabbit IgG-HRP or goat anti-mouse IgG-HRP. (D and F) Densitometry analysis plots of the kinetics
466 of the relative levels of P-LIMK1/total LIMK1 (D) or P-cofilin/total cofilin (F) after being
467 normalized by β-actin. PRRSV, porcine reproductive and respiratory syndrome virus; LIMK1,
468 LIM domain kinase 1; PBS, phosphate-buffered saline; SDS-PAGE, sodium dodecyl sulfate-
469 polyacrylamide gel electrophoresis; HRP, horseradish peroxidase; P-cofilin, phospho-cofilin; P-
470 LIMK1, phospho-LIMK1; siRNA, small interfering RNA; MOI, multiplicity of infection; mpi,

471

472

473

474

475

476

477

478

479

480

minute post-infection.

## 481 Supporting information

###### 482 S1. Uncropped western blot images.

<https://www2.cloud.editorialmanager.com/pone/download.aspx?id=38216030&guid=bf3ab278-f8a1-4c1e-8f5b-3a44bd579e9b&scheme=1>Figure1	[Click here to access/download;Figure;FIG1_Final.pptx](https://www2.cloud.editorialmanager.com/pone/download.aspx?id=38216030&guid=bf3ab278-f8a1-4c1e-8f5b-3a44bd579e9b&scheme=1)

**Figure 1.**

# A B

**DMSO EIPA Rottlerin	NC siPKCδ**

N protein N protein

β-Actin β-Actin

# C D

### DMSO Rottlerin EIPA

Figure2

**Figure 2.**

**B	C** [Click here to access/download;Figure;FIG2_Final.pptx](https://www2.cloud.editorialmanager.com/pone/download.aspx?id=38216031&guid=7c65be6f-0ada-4272-86d7-b6e255333853&scheme=1)[<https://www2.cloud.editorialmanager.com/pone/download.aspx?id=38216031&guid=7c65be6f-0ada-4272-86d7-b6e255333853&scheme=1>](https://www2.cloud.editorialmanager.com/pone/download.aspx?id=38216031&guid=7c65be6f-0ada-4272-86d7-b6e255333853&scheme=1)

# E

**D**

**A**

Figure3	[Click here to access/download;Figure;FIG3_Final2.pptx](https://www2.cloud.editorialmanager.com/pone/download.aspx?id=38216032&guid=0c02266e-ac76-4816-a96c-f852eeea5465&scheme=1)[<https://www2.cloud.editorialmanager.com/pone/download.aspx?id=38216032&guid=0c02266e-ac76-4816-a96c-f852eeea5465&scheme=1>](https://www2.cloud.editorialmanager.com/pone/download.aspx?id=38216032&guid=0c02266e-ac76-4816-a96c-f852eeea5465&scheme=1)

**Figure 3.**

**A**

N protein

Total LIMK1 Total cofilin

β-Actin

**Relative (N protein)**

**MOCK	siNC siLIMK1 sicofilin**

# B

**C	MOCK	DMSO	Rottlerin	D**

mpi	60	5	15	30	120	5	15	30	120

P-LIMK1

Total LIMK1

β-Actin

**MOCK	DMSO	Rottlerin**

**E**

**F**

mpi	60 5 15 30 60 90 120 5 15 30 60 90 120

P-cofilin (Ser3) Total cofilin

β-Actin

Figure 1_Revised	[Click here to access/download;Figure;FIG1.tif](https://www2.cloud.editorialmanager.com/pone/download.aspx?id=38206243&guid=ab44e609-b3cd-426f-8a5b-32e4f0a4e3c8&scheme=1)[<https://www2.cloud.editorialmanager.com/pone/download.aspx?id=38206243&guid=ab44e609-b3cd-426f-8a5b-32e4f0a4e3c8&scheme=1>](https://www2.cloud.editorialmanager.com/pone/download.aspx?id=38206243&guid=ab44e609-b3cd-426f-8a5b-32e4f0a4e3c8&scheme=1)

Figure 2_Revised	[Click here to access/download;Figure;FIG2.tif](https://www2.cloud.editorialmanager.com/pone/download.aspx?id=38206244&guid=5dae5fb4-0ac1-4be0-9f52-05a18ef2fdf3&scheme=1)[<https://www2.cloud.editorialmanager.com/pone/download.aspx?id=38206244&guid=5dae5fb4-0ac1-4be0-9f52-05a18ef2fdf3&scheme=1>](https://www2.cloud.editorialmanager.com/pone/download.aspx?id=38206244&guid=5dae5fb4-0ac1-4be0-9f52-05a18ef2fdf3&scheme=1)

Figure 3_Revised	[Click here to access/download;Figure;FIG3.tif](https://www2.cloud.editorialmanager.com/pone/download.aspx?id=38206245&guid=ac96e867-27f1-4172-b32c-16fc2b23af4d&scheme=1)[<https://www2.cloud.editorialmanager.com/pone/download.aspx?id=38206245&guid=ac96e867-27f1-4172-b32c-16fc2b23af4d&scheme=1>](https://www2.cloud.editorialmanager.com/pone/download.aspx?id=38206245&guid=ac96e867-27f1-4172-b32c-16fc2b23af4d&scheme=1)

Supporting Information

#### [Click here to access/download](https://www2.cloud.editorialmanager.com/pone/download.aspx?id=38206242&guid=7f1b8b71-4e12-49e8-badb-0eba61793e91&scheme=1)

[**Supporting Information** S1_raw_images_uncropped WB.pdf](https://www2.cloud.editorialmanager.com/pone/download.aspx?id=38206242&guid=7f1b8b71-4e12-49e8-badb-0eba61793e91&scheme=1)

Revised Manuscript with Track Changes

###### 1 Rottlerin inhibits macropinocytosis of Porcine Reproductive and

2	**Respiratory Syndrome Virus through the PKCδ-Cofilin signaling**

3	**pathway**

4

5	Yeonglim Kang^a^, Jong-Chul Choi^a^, Joong-Bok Lee^a^, Seung-Yong Park^a^, and Changin Oh^b*^

6

7 a*Laboratory of Infectious Diseases, College of Veterinary Medicine, Konkuk University, Seoul*
8 *~~05029~~, Republic of Korea*
9 b*Department of Genetics, Yale University School of Medicine, New Haven, Connecticut~~T 06520-~~*
10 *~~8005~~, United States of America*

**Formatted:** Font:

11

12 ***Corresponding author~~:~~** ~~Changin Oh~~

**Formatted:** Font:

13 ~~Department of Genetics, Yale University School of Medicine, New Haven, CT 06520-8005, USA~~
14 E-mail: [changin.oh@yale.edu](mailto:changin.oh@yale.edu) (CO)

15

16

17

18

19

**Formatted:** Font:

20	**Abstract**

**Formatted:** 제목 11, Line spacing: single

21 Rottlerin exerts antiviral activity against various enveloped viruses, yet the mechanism by which
22 it inhibits viral replication and the associated signaling pathways remains unclear. Here, we
23 investigated the mechanisms for the antiviral effects of Rottlerin against Porcine Reproductive and
24 Respiratory Syndrome Virus (PRRSV) in vitro. We demonstrate that PRRSV enters host cells via
25 macropinocytosis. Rottlerin, a PKCδ inhibitor, partially inhibits PRRSV entry by decreasing actin
26 polymerization. LIM domain kinase 1 (LIMK1) is essential for PRRSV replication, and PRRSV
27 infection-induced cofilin activation, which was reversed by rottlerin treatment. Our findings
28 suggest that a subset of PRRSV utilizes PKCδ-mediated actin dynamics to enter cells via
29 macropinocytosis, and that rottlerin is a potential antiviral molecule targeting this entry pathway.

30

31 **Keywords**
32 PKCδ, rottlerin, PRRSV, antivirals, macropinocytosis

33

**Formatted:** Highlight

**Formatted:** 제목 11, Line spacing: single

34

**Introduction**

35 Macropinocytosis is an endocytic process that leads to the internalization of fluids, extracellular
36 particles, and membranes into large vacuoles (1). Activation of actin and microfilaments induces
37 macropinocytosis through the formation of lamellipodia, membrane ruffles, and blebs on the
38 cellular periphery (2). Various viral families, such as human immunodeficiency virus (HIV),
39 vaccinia virus, and adenovirus, and various types of bacteria take advantage of macropinocytosis
40 as a direct means of cellular internalization or an indirect mechanism to assist the penetration of
41 particles that have entered by other forms of endocytosis (2). Thus, blocking macropinocytosis
42 may be a potential strategy for controlling broad-spectrum viral infections.
43 Porcine reproductive and respiratory syndrome virus (PRRSV) is an enveloped positive-sense
44 ssRNA virus belonging to the family Arteriviridae and order Nidovirales (3, 4). PRRSV infection
45 causes reproductive impairments in sows and respiratory diseases in pigs of all ages, resulting in
46 a critical economic impact on the global swine industry (5, 6). It has been reported that PRRSV
47 enters into host cells via low pH-dependent, clathrin-mediated endocytosis, utilizing CD163 as an
48 essential receptor (7-9). A recent paper claims that PRRSV uses macropinocytosis as an alternative
49 entry route (10). However, Additional evidence and detailed mechanisms underlying PRRSV-
50 induced macropinocytosis are necessary to support this novel finding. We recently demonstrated
51 that rottlerin blocks PRRSV endocytosis by inhibiting PRRSV-induced protein kinase C delta
52 (PKCδ) phosphorylation (11), suggesting that inhibition of PKCδ phosphorylation suppresses one
53 or more PRRSV endocytosis routes. Thus, we investigated the antiviral mechanisms of rottlerin
54 against PRRSV, especially how rottlerin blocks PRRSV endocytosis.
55 Here, we demonstrated that PRRSV enters MARC-145 cells via macropinocytosis during infection,

**Formatted:** Highlight

**Formatted:** 제목 11, Line spacing: single

**Formatted:** Highlight

**Formatted:** 제목 21, Line spacing: single

**Formatted:** Highlight

56 which is inhibited by rottlerin treatment, by decreasing actin polymerization. PRRSV
57 macropinocytosis is mediated by the PKCδ-cofilin pathway and LIM domain kinase 1 (LIMK1).
58 The results of the present study provide a possible explanation for the antiviral effect of rottlerin
59 on other viruses and contribute to an understanding of the PRRSV entry mechanism.

60

61

**Materials and Methods**

62

**Viruses**

63 The PRRSV FL12 strain used in this study was rescued from the pCMV-FL12/24 plasmid that was
64 kindly provided by Professor Won-Il Kim (12). Briefly, semi-confluent MARC-145 cells were
65 transfected with pCMV-FL12/24 and the ViaFect reagent (Promega, Madison, WI, USA) in a 1:3
66 ratio. After 72 hours of transfection, cell supernatants were harvested and passaged into MARC-
67 145 cells. Viral titers were measured using quantitative reverse transcription–polymerase chain
68 reaction (qRT-PCR) and titration. Viral stocks were stored at −80 ℃. Viruses were concentrated
69 by centrifugation using a Beckman SW-41 Ti rotor at 160,000 × *g* at 4 ℃ for 4 hours through a
70 discontinuous sucrose gradient consisting of 1 mL of 25%, 35%, 45%, 55%, and 65% sucrose. The
71 35%–45% fraction was dialyzed and titrated for future experiments.

72

73

**Immunofluorescence assay and confocal microscopy**

74 Approximately 2.4 × 10^4^ MARC-145 cells per well were seeded on an eight-chamber slide. On
75 the following day, MARC-145 cells were preincubated with rottlerin (5 μM) or 5-[*N*-ethyl-*N*-

**Formatted:** Highlight

**Formatted:** 제목 21, Line spacing: single

76 isopropyl] amiloride (EIPA) (50 μM) (Tocris, Bristol, UK) at 37 °C for 2 hours or left untreated.
77 For the viral endocytosis assay, cells were infected with PRRSV FL12 at a multiplicity of infection
78 (MOI) of 50 for 1 hour. Cells were washed and treated with proteinase K to remove extracellular
79 viruses for 45 minutes at 4°C. For the dextran uptake assay, cells were inoculated with CF®640R
80 dextran (10,000 MW) (Biotium, Fremont, CA, USA) with or without PRRSV FL12 (MOI = 50),
81 incubated for 1 hour, and washed with 3 M sodium acetate buffer (pH 5.2). The cells were fixed
82 with 4% paraformaldehyde for 15 minutes and permeabilized with Tris-buffered saline (TBS)
83 containing 0.2% Triton X-100 at room temperature (RT) for 10 minutes. The cells were then
84 probed as previously described (13) with minor modifications. CF®640R phalloidin (Biotium,
85 Fremont, CA, USA) was used to label the F-actin. An anti-PRRSV nucleocapsid (N) monoclonal
86 antibody (1:200) (Median Diagnostics, Chuncheon, Korea) was used to label the viral particles.
87 The cells were scanned using an EVOS FL Cell Imaging System (Thermo Fisher Scientific,
88 Waltham, MA, USA) or an LSM 900 with Airyscan 2 (Zeiss, Oberkochen, Germany). The images
89 were processed, and the corrected total cell fluorescence was calculated using ImageJ software
90 (NIH, Bethesda, MD, USA).

91

92

**RNA interference**

93 To silence PKCδ, LIMK1, and cofilin expression, MARC-145 cells were transfected with small
94 interfering RNAs (siRNAs) targeting PKCδ, LIMK1, and cofilin, respectively (14) using
95 Lipofectamine 3000 (Thermo Fisher Scientific) according to the manufacturer’s instructions, and
96 the cells were incubated at 37 °C for 24 hours.

97

**Formatted:** Highlight

**Formatted:** 제목 21, Line spacing: single

**Formatted:** Highlight

**Formatted:** 제목 21, Line spacing: single

98

**Western blot**

99 To test the effects of rottlerin on the phosphorylation of LIMK1 and cofilin, confluent MARC-145
100 cells were treated with rottlerin (5 μM) or EIPA (50 μM), respectively, for 2 hours at 37 ℃. The
101 cells were inoculated with PRRSV FL12 (MOI = 1) and incubated for the indicated times. Cells
102 were lysed using mammalian protein extraction reagent (Thermo Fisher Scientific) with a Halt
103 protease phosphatase inhibitor cocktail (Thermo Fisher Scientific). Cell lysates were resolved
104 using sodium dodecyl sulfate-polyacrylamide gel electrophoresis (Bolt 4%–12% Bis-Tris gel;
105 Invitrogen, Waltham, MA, USA) and transferred onto nitrocellulose membranes (Komabiotech,
106 Seoul, Korea). The membranes were blocked with 5% skim milk in TBS with Tween 20 (TBS-T)
107 for 30 minutes at RT and probed with rabbit anti-cofilin (P-cofilin, Ser3) monoclonal antibody
108 (1:1,000) (Cell Signaling Technology, Danvers, MA, USA), mouse anti-cofilin monoclonal
109 antibody–horseradish peroxidase (HRP) (1:1,000) (Santa Cruz Biotechnology, Dallas, TX, USA),
110 rabbit anti-LIMK1/2 (P-LIMK1, Thr508/505) polyclonal antibody (1:1,000) (BioVision, Waltham,
111 MA, USA), rabbit anti-LIMK1 polyclonal antibody (1:1,000) (Cell Signaling Technology), or
112 mouse anti-β-actin monoclonal antibody-HRP (1:50,000) (Abcam, Cambridge, UK) overnight at
113 4 ℃. After five washes with TBS-T, the membranes were incubated with HRP-conjugated anti-
114 rabbit IgG (Komabiotech) (1:5,000) for 1 hour at RT. Proteins were visualized using Clarity
115 Western ECL Substrate (Bio-Rad, Hercules, CA, USA) and detected using FUSION Solo S (Vilber,
116 Collégien, France). The density of the protein bands was measured using ImageJ software (NIH)
117 after subtracting the density of the β-actin bands.

118

119

**qRT-PCR and virus titration**

**Formatted:** Highlight

**Formatted:** 제목 21, Line spacing: single

**Formatted:** Font:

120 Viral RNA was extracted from the supernatants of PRRSV-infected cells using an RNeasy Mini
121 Kit (QIAGEN, Hilden, Germany), according to the manufacturer's recommendations. Viral RNA
122 levels were quantified using the RNA UltraSense One-Step Quantitative RT-PCR System
123 (Invitrogen) with the LightCycler 96 System (Roche, Basel, Switzerland), as previously described
124 (15). The measured cycle threshold (CT) values were converted to RNA copy numbers and tissue
125 culture infective dose 50% (TCID50) equivalent/mL using a standard curve generated from CT
126 values plotted against the corresponding TCID50/mL. The viral titer (TCID50/mL) of the collected
127 samples was measured using the Reed–Muench method, as previously described (16).

128

129

**Statistical analyses**

130 All data were analyzed using IBM SPSS Statistics 25 (IBM Corp., Armonk, NY, USA) and
131 GraphPad Prism 6 (GraphPad Software, San Diego, CA, USA) using a nonparametric model
132 (Kruskal–Wallis followed by Mann–Whitney). All data are presented as the mean ± standard
133 deviation, and *, *p*-value <0.0332~~5~~; **, *p*-value <0.0021; ***, *p*-value <0.0002; ****, *p*-value
134 <0.0001~~1~~ were considered significant.

135

136

137

138

139

**Formatted:** Highlight

**Formatted:** 제목 11, Line spacing: single

140

**Results**

###### 141 Rottlerin inhibits PRRSV endocytosis in MARC-145 cells

142 We previously reported that rottlerin inhibits PRRSV endocytosis(11), but the detailed mechanism
143 of its antiviral activity and involvement of other PKCδ related cellular molecules is unknown. To
144 investigate the role of the PKCδ and elucidate the antiviral mechanism of rottlerin during PRRSV
145 infection, we examined PRRSV replication in the presence of rottlerin in MARC-145 cells. The
146 cells were pre-treated with rottlerin (5 μM) or EIPA (50 μM), or subjected to PKCδ silencing,
147 followed by infection with PRRSV FL12 (MOI= 1). Subsequently, viral replication was evaluated
148 using three distinct parameters at 48 hours post-infection (hpi): PRRSV RNA synthesis, PRRSV
149 nucleocapsid (N) protein expression, and infectious virus production. Consistent with our previous
150 report, both rottlerin and siRNA treatment significantly inhibited viral RNA synthesis (Fig. 1A),
151 expression of the N protein (Fig. 1B), and production of infectious progeny viruses (Fig. 1C).
152 To further determine whether rottlerin blocks PRRSV endocytosis, we used immunofluorescence
153 to observe the localization of PRRSV particles in infected MARC-145 cells after treatment with
154 rottlerin or RIPA. The cells were washed with sodium acetate buffer (pH 5.2) to eliminate the
155 extracellular viruses. As expected, confocal microscopy images of dimethyl sulfoxide (DMSO)-
156 treated cells showed localization of PRRSV to the cytoplasm with a puncta shape (Fig. 1D, left),
157 whereas rottlerin-treated cells showed the absence of a signal in the cytoplasm (Fig. 1D, middle),
158 similar to EIPA treated cells (Fig. 1D, right). This indicates that rottlerin treatment impaired
159 PRRSV endocytosis. These results are consistent with our previous findings showing that rottlerin
160 treatment suppressed PRRSV replication in cells and pigs by inhibiting PRRSV endocytosis (11).

**Formatted:** Highlight

**Formatted:** 제목 21, Line spacing: single

161

**Rottlerin inhibited macropinocytosis of PRRSV by decreasing actin**

162	**polymerization**

163 Due to the inhibition of PRRSV replication and endocytosis by EIPA treatment (Fig. 1), a selective
164 macropinocytosis inhibitor (17), we investigated whether PRRSV entry occurs via
165 macropinocytosis and if rottlerin inhibits PRRSV macropinocytosis. We co-incubated MARC-145
166 cells for an hour with fluorescence-labeled CF®640R dextran (10,000 MW), which is known to
167 enter cells via macropinocytosis (18, 19) (Fig. 2A). In DMSO-treated cells, dextran signals were
168 observed in the cytoplasm, suggesting macropinocytosis of dextran (Fig. 2A, top). In contrast,
169 rottlerin or EIPA-treated cells showed reduced dextran signals around the nucleus (Fig. 2A, middle
170 and bottom), indicating that rottlerin also inhibits macropinocytosis.
171 Next, PRRSV and dextran were co-inoculated into DMSO, rottlerin, or EIPA-treated cells. In
172 DMSO-treated cells, signals for both dextran and PRRSV were observed around the nucleus with
173 some colocalization (Fig. 2B, top row). This suggests that during the endocytosis of the virus and
174 dextran, some of them enter together through macropinocytosis. In contrast, either rottlerin or EIPA
175 treatment significantly decreased the number of both signals in cells with significantly less co-
176 localization between them (Fig. 2C, Pearson’s coefficient: DMSO, 0.43 vs. rottlerin, 0.07). These
177 results suggest that rottlerin inhibited PRRSV macropinocytosis in MARC-145 cells.
178 Among the several characteristics of macropinocytosis, actin polymerization is a key factor for
179 macropinocytosis-specific uptake (10, 20). Therefore, we tested whether rottlerin reduced actin
180 polymerization during PRRSV macropinocytosis similar to EIPA. Actin dynamics were observed
181 using fluorescently labeled phalloidin, a specific ligand of F-actin. DMSO-treated cells after

**Formatted:** Highlight

**Formatted:** 제목 21, Line spacing: single

182 PRRSV infection showed multiple pseudopods projecting from the plasma membrane into the
183 outer space (white arrow) (Fig. 2D, top row). Interestingly, rottlerin or EIPA treatment caused the
184 disappearance of these projections (Fig. 2D, middle and bottom rows). Quantitative analysis
185 showed that cells with proper projections were significantly decreased when treated with rottlerin
186 (*p*-value < 0.05) and EIPA (*p*-value < 0.01) compared with the DMSO-treated cells (Fig. 2E),
187 confirming that rottlerin inhibited PRRSV macropinocytosis by reducing actin polymerization.

188

189

**Rottlerin inhibits PRRSV endocytosis through the PKCδ-cofilin**

###### 190 signaling pathway

191 PKCδ regulates actin dynamics during macropinocytosis via downstream effectors such as cofilin,
192 slingshot phosphatase homolog 1, and LIMK1(21). Cofilin regulates the dynamics of the actin
193 cytoskeleton by binding to an actin filament(22, 23). LIMK1 directly phosphorylates cofilin at Ser-
194 3, which inactivates it (21, 24, 25). To evaluate the involvement of cofilin and LIMK1 in PRRSV
195 macropinocytosis, cofilin- or LIMK1-silenced MARC-145 cells were used to measure PRRSV
196 replication. Transfection of siRNAs targeting cofilin or LIMK1 effectively knocked down the
197 expression of each protein in the cells (Fig. 3A). Interestingly silencing LIMK1 resulted in
198 decreased expression of cofilin.
199 Next, we investigated the effect of cofilin or LIMK1 silencing on PRRSV replication by measuring
200 viral titer and expression of the N protein in siRNA-treated cells after PRRSV infection. The N
201 protein blot showed that silencing LIMK1 significantly inhibited N protein synthesis with the
202 relative intensity of the band being 0.36 compared to treatment with siNC (Fig. 3A, Relative N
203 protein intensity:1.00). Silencing Cofilin resulted in an intensity of 0.84 for the N protein band,
204 but this effect was not as significant as that observed with LIMK1 silencing. Consistent with the
205 western blot results, the silencing of LIMK1 notably reduced the viral titer by 11-fold compared
206 to siNC-treated cells (Fig. 3B)., ~~whereas~~ C~~c~~ofilin silencing also ~~did not~~ significantly ~~impact~~
207 decreased the viral titer but less than that of siLIMK1. These results suggest that both LIMK1 and
208 cofilin serves as a host dependency factor essential for PRRSV infection.
209 To further investigate how macropinocytosis is regulated by rottlerin following PRRSV infection,
210 we monitored the activity of cofilin or LIMK1 in PRRSV-infected cells. Since the activities of
211 LIMK1 and cofilin are regulated by phosphorylation, we examined the levels of phosphorylated
212 LIMK1 and cofilin (p~~P~~-LIMK1 and p-cofilin) as well as total LIMK1 and cofilin using
213 immunoblots. As shown in Fig. 3C and D, the levels of p~~P~~-LIMK1 after PRRSV infection with
214 DMSO transiently increased immediately after PRRSV infection (15 mpi), slightly
215 dephosphorylated (30 mpi), and then re-phosphorylated at a later time point (120 mpi). In contrast,
216 rottlerin treatment rapidly attenuated the virus-induced p~~P~~-LIMK1, and the p~~P~~-LIMK1 levels in
217 rottlerin-treated cells were returned similar to Mock-treated cells at 30, and 120 mpi (Fig. 3C and
218 D). Similar levels of total LIMK1 after DMSO or rottlerin treatment suggest that the changes in
219 p~~P~~- LIMK1 were not due to a change in total LIMK1. These results suggest that PRRSV infection
220 mediates transient activation of LIMK1 at an early stage of infection through PKCδ, which was
221 rapidly attenuated by rottlerin.
222 PRRSV infection gradually decreased p~~P~~-cofilin levels in DMSO-treated cells, and a low level of
223 p~~P~~-cofilin was maintained up to 120 mpi (Fig. 3E and F). In contrast, rottlerin treatment led to
224 increase p~~P~~- cofilin. The levels of p~~P~~-cofilin in rottlerin-treated cells began to increase at 15 mpi

**Formatted:** Highlight

**Formatted:** 제목 11, Line spacing: single

225 and continued to rise until 120 mpi (Fig. 3E and F). Quantitative analysis of the blot bands showed
226 that rottlerin treatment significantly increased p~~P~~-cofilin levels at 90 and 120 mpi compared with
227 DMSO treatment (Fig. 3F). These results demonstrate that PRRSV infection decreases P-cofilin
228 levels, whereas rottlerin treatment reverses this effect. Overall, the findings suggest that rottlerin
229 regulates LIMK1 and cofilin signaling pathways, which may contribute to the mechanism by
230 which rottlerin inhibits PRRSV-induced macropinocytosis**.**

231

**Discussion**

232 In this study, our microscopic and biochemical analyses demonstrated that rottlerin treatment
233 inhibited PRRSV replication by decreasing viral endocytosis in vitro, which resulted in the
234 suppression of viral RNA synthesis, N protein expression, and infectious virus production (Fig. 1).
235 We also showed that PRRSV entered MARC-145 cells via macropinocytosis, and rottlerin blocked
236 it by decreasing actin polymerization (Fig. 2). LIMK1 acts as a host dependency factor crucial for
237 PRRSV infection. Rottlerin reversed PRRSV-induced activation of cofilin (Fig. 3). These findings
238 provide a possible explanation for one of the antiviral mechanisms of rottlerin at the early stages
239 of virus infection, i.e., rottlerin blocks macropinocytosis of viruses by decreasing actin
240 polymerization via the PKCδ-cofilin signaling pathway. In the case of HIV1 and Zika virus,
241 rottlerin inhibits an early stage of their replication (26, 27) and they are known to enter cells via
242 actin-related entry pathways, indicating that rottlerin could inhibit their entry through a similar
243 mechanism (28-30).
244 Our results demonstrated that macropinocytosis serves as an alternative route for PRRSV to
245 infect host cells. Our findings match with a recent study that PRRSV infects host cells through
246 macropinocytosis, involving T-cell immunoglobulin and mucin domain proteins as well as CD163
247 (10). We further hypothesize that PRRSV possibly enters cells via at least two distinct pathways:
248 clathrin-mediated endocytosis and macropinocytosis. Few in vitro assay results support the
249 hypothesis that a single treatment with chlorpromazine, a clathrin lattice polymerization inhibitor,
250 or rottlerin partially blocked PRRSV replication (Fig. 1C), but the combination of both inhibitors
251 almost completely inhibited PRRSV replication (10). Understanding the potential PRRSV entry
252 routes is crucial for the clinical application of antiviral drugs. To achieve an effective anti-PRRSV
253 treatment, a combination of inhibitors targeting both entry routes, rather than a single treatment
254 with either route inhibitor, is necessary.
255 Our results provide insights into how PRRSV induces macropinocytosis through the PKCδ-
256 LIMK1-cofilin signaling pathway to infect host cells. Although macropinocytosis signaling
257 mechanisms have been extensively studied over the last decade (2, 21), the PRRSV-mediated
258 signaling pathway of macropinocytosis remains unclear. Generally, to induce macropinocytosis by
259 cofilin and LIMK1, the binding of the active form of cofilin (dephosphorylated at Ser3)
260 depolymerizes actin by severing the actin filaments, which finally induces lamellipodium
261 formation and macropinocytosis(31). However, filament severing also results in actin
262 polymerization, depending on the relative amount of actin monomers and free barb ends (32). LIM
263 kinases directly phosphorylate cofilin, which allows the precise regulation of actin dynamics (33).
264 LIMK1 is known to not only phosphorylate cofilin but also directly bind F-actin to induce actin
265 depolymerization and membrane ruffling (34). In the case of PRRSV, we propose that viral
266 infection activates cofilin to increase lamellipodium formation for macropinocytosis, which
267 increases viral uptake from the extracellular space (21). Rottlerin treatment inhibits this activation
268 by blocking upstream PKCδ(11). Our microscopic data is in line with this model (Fig. 2).
269 Our results regarding LIMK1 phosphorylation cannot be fully explained by the standard cofilin-
270 LIMK1 pathway. In our study, PRRSV infection induced LIMK1 phosphorylation (Fig. 3C and
271 D), which is contrary to what would be expected based on our microscopic and cofilin
272 phosphorylation results. Typically, phosphorylated LIMK1 adds a phosphate group to cofilin,
273 thereby inactivating cofilin and stabilizing actin filaments. Considering all our results, LIMK1
274 may play an additional role in this virus entry process. It is possible that LIMK1 not only
275 phosphorylates cofilin but also precisely regulates this pathway. Previous research shows LIMK1
276 is involved in the microtubule destabilization(35), suggesting LIMK1 may directly coordinate
277 microtubules and actin cytoskeleton. Another research also supports this notion, indicating that
278 pharmacological inhibition of LIMK1 does not stimulate macropinocytosis(21). Even if LIMK1
279 did not follow the standard pathway, it appears that LIMK1 is a key regulator employed by PRRSV
280 to control macropinocytosis. These results are consistent with previous studies showing that
281 PRRSV replication requires PKCδ (36). HIV binding to CD4+ T cells initiates transient activation
282 of LIMK and actin polymerization (37). Small-molecule inhibitors of LIM kinases have been
283 reported to inhibit HIV1 (38). Further research is needed to study the mechanisms underlying
284 virus-induced macropinocytosis.
285 Rottlerin is a secondary metabolite isolated from *Mallotus philippinensis*, which has been
286 traditionally used to treat tapeworm, scabies, and herpetic ringworm infections in India for several
287 centuries (39). Rottlerin is a selective inhibitor of PKCδ (40, 41), but recent studies have reported
288 that rottlerin inhibits multiple kinases (42-44). In our study, PKCδ inhibition of rottlerin is highly
289 likely to be a major blocking mechanism of PRRSV macropinocytosis, rather than other
290 mechanisms, because silencing of PKCδ decreased PRRSV replication (Fig. 1), and we have
291 previously shown that rottlerin treatment dephosphorylates PKCδ induced by PRRSV infection

**Formatted:** 제목 11, Line spacing: single

292 (11). Interestingly, several other studies have demonstrated the PKCδ-independent antiviral effects
293 of rottlerin (43, 44). Ojha et al. recently showed that rottlerin inhibits La Crosse virus trafficking
294 from the Golgi apparatus to the trans-Golgi vesicles, which may be related to decreased cellular
295 ATP levels induced by rottlerin (43, 45). These results suggest that rottlerin can block various steps
296 of viral replication, thus strengthening its antiviral potency. Thus, an intensive mechanistic study
297 of the antiviral effects of rottlerin against various viruses is required.
298 In conclusion, the current study demonstrates that PRRSV utilizes macropinocytosis to infect host
299 cells. Rottlerin blocks macropinocytosis of PRRSV related to PKCδ-cofilin signaling pathways by
300 reducing actin polymerization. Our results suggest that rottlerin is a potential antiviral drug
301 targeting macropinocytosis and its associated signaling molecules.

302

303

##### 304 ~~Clinical Perspectives~~

305	**~~Point 1.~~** ~~Rottlerin inhibited PRRSV replication by reducing viral endocytosis.~~

306	**~~Point 2.~~** ~~Rottlerin suppresses PRRSV-induced macropinocytosis and actin polymerization through~~

307	~~the PKCδ-cofilin signaling pathway.~~

308	**~~Point 3.~~** ~~Rottlerin is a potential broad-spectrum antiviral drug targeting macropinocytosis.~~

309

**Formatted:** Highlight

**Formatted:** 제목 11, Line spacing: single

**Formatted:** Highlight

**Formatted:** Font:

**Formatted:** 제목 11, Line spacing: single

**Formatted:** 제목 11, Line spacing: single

**Formatted:** 제목 11, Line spacing: single

##### 310 ~~Data availability statement~~

311	~~All data needed to evaluate the conclusions in the paper are present in the paper.~~

##### 312 ~~Author Contribution~~

313	~~Y.K. and C.O. designed research; Y.K., I.C., and J.C. performed research; Y.K., J.C., and C.O.~~

314	~~analyzed data; Y.K., J.L., S.P., and C.O. wrote the paper.~~

##### 315 ~~Declaration of competing interest~~

316	~~The authors declare no competing interests.~~

317

318

319

**Acknowledgment**

320

~~This research was supported by the Korea Institute of Planning and Evaluation for Technology in~~

321 ~~Food, Agriculture, and Forestry (IPET) through the Agriculture, Food, and Rural Affairs~~
322 ~~Convergence Technologies Program for Educating the Creative Global Leader Program, funded~~
323 ~~by the Ministry of Agriculture, Food, and Rural Affairs (MAFRA) (320005-04).~~ We are deeply
324 grateful to the KU Research Center for Zoonosis for providing advice on the design and progress
325 of this study.

326

327	**References**

**Formatted:** 제목 11, Line spacing: single

328

329

330

331

332

333

334

335

336

337

338

339

340

341

342

343

344

345

346

347

348

349

350

351

352

353

354

355

356

357

358

359

360

361

362

363

364

365

366

367

368

369

370

371

372

373

374

375

376

377

1. Zeineddine R, Yerbury JJ. The role of macropinocytosis in the propagation of protein aggregation associated with neurodegenerative diseases. Front Physiol. 2015;6:277.
2. Mercer J, Helenius A. Virus entry by macropinocytosis. Nat Cell Biol. 2009;11(5):510-20.

**Formatted:** Font: (Default) Times New Roman

3. Cavanagh D. Nidovirales: a new order comprising Coronaviridae and Arteriviridae. Arch Virol. 1997;142(3):629-33.
4. Conzelmann KK, Visser N, Van Woensel P, Thiel HJ. Molecular characterization of porcine reproductive and respiratory syndrome virus, a member of the arterivirus group. Virology. 1993;193(1):329 -39.
5. Meulenberg JJ, Hulst MM, de Meijer EJ, Moonen PL, den Besten A, de Kluyver EP, et al. Lelystad virus, the causative agent of porcine epidemic abortion and respiratory syndrome (PEARS), is related to LDV and EAV. Virology. 1993;192(1):62-72.
6. Neumann EJ, Kliebenstein JB, Johnson CD, Mabry JW, Bush EJ, Seitzinger AH, et al. Assessment of the economic impact of porcine reproductive and respiratory syndrome on swine production in the United States. J Am Vet Med Assoc. 2005;227(3):385-92.
7. Nauwynck HJ, Duan X, Favoreel HW, Van Oostveldt P, Pensaert MB. Entry of porcine reproductive and respiratory syndrome virus into porcine alveolar macrophages via receptor-mediated endocytosis. J Gen Virol. 1999;80 ( Pt 2):297-305.
8. Calvert JG, Slade DE, Shields SL, Jolie R, Mannan RM, Ankenbauer RG, et al. CD163 expression confers susceptibility to porcine reproductive and respiratory syndrome viruses. J Virol. 2007;81(14):7371-9.
9. Shi C, Liu Y, Ding Y, Zhang Y, Zhang J. PRRSV receptors and their roles in virus infection. Arch Microbiol. 2015;197(4):503-12.
10. Wei X, Li R, Qiao S, Chen XX, Xing G, Zhang G. Porcine Reproductive and Respiratory Syndrome Virus Utilizes Viral Apoptotic Mimicry as an Alternative Pathway To Infect Host Cells. J Virol. 2020;94(17).
11. Kang YL, Oh C, Ahn SH, Choi JC, Choi HY, Lee SW, et al. Inhibition of endocytosis of porcine reproductive and respiratory syndrome virus by rottlerin and its potential prophylactic administration in piglets. Antiviral Res. 2021;195:105191.
12. Shabir N, Khatun A, Nazki S, Kim B, Choi EJ, Sun D, et al. Evaluation of the Cross-Protective Efficacy of a Chimeric Porcine Reproductive and Respiratory Syndrome Virus Constructed Based on Two Field Strains. Viruses. 2016;8(8).
13. Zhu L, Bi J, Zheng L, Zhao Q, Shu X, Guo G, et al. In vitro inhibition of porcine reproductive and respiratory syndrome virus replication by short antisense oligonucleotides with locked nucleic acid modification. BMC Vet Res. 2018;14(1):109.
14. Choi K, Ryu SW, Song S, Choi H, Kang SW, Choi C. Caspase-dependent generation of reactive oxygen species in human astrocytoma cells contributes to resistance to TRAIL-mediated apoptosis. Cell Death Differ. 2010;17(5):833-45.
15. Wernike K, Hoffmann B, Dauber M, Lange E, Schirrmeier H, Beer M. Detection and typing of highly pathogenic porcine reproductive and respiratory syndrome virus by multiplex real-time rt-PCR. PLoS One. 2012;7(6):e38251.
16. Kim WI, Lee DS, Johnson W, Roof M, Cha SH, Yoon KJ. Effect of genotypic and biotypic differences among PRRS viruses on the serologic assessment of pigs for virus infection. Vet Microbiol. 2007;123(1 -3):1-14.
17. Jayashankar V, Edinger AL. Macropinocytosis confers resistance to therapies targeting cancer anabolism. Nat Commun. 2020;11(1):1121.
18. Falcone S, Cocucci E, Podini P, Kirchhausen T, Clementi E, Meldolesi J. Macropinocytosis: regulated coordination of endocytic and exocytic membrane traffic events. J Cell Sci. 2006;119(Pt 22):4758-69.
19. Nakase I, Hirose H, Tanaka G, Tadokoro A, Kobayashi S, Takeuchi T, et al. Cell-surface accumulation of flock house virus-derived peptide leads to efficient internalization via macropinocytosis. Mol Ther. 2009;17(11):1868- 76.
20. Commisso C, Flinn RJ, Bar-Sagi D. Determining the macropinocytic index of cells through a quantitative image-based assay. Nat Protoc. 2014;9(1):182-92.
21. Singla B, Lin HP, Ghoshal P, Cherian-Shaw M, Csanyi G. PKCdelta stimulates macropinocytosis via activation of SSH1-cofilin pathway. Cell Signal. 2019;53:111-21.

378

379

380

381

382

383

384

385

386

387

388

389

390

391

392

393

394

395

396

397

398

399

400

401

402

403

404

405

406

407

408

409

410

411

412

413

414

415

416

417

418

419

420

421

422

423

424

425

426

427

428

429

430

431

432

22. Ghosh M, Song X, Mouneimne G, Sidani M, Lawrence DS, Condeelis JS. Cofilin promotes actin polymerization and defines the direction of cell motility. Science. 2004;304(5671):743-6.
23. Hotulainen P, Paunola E, Vartiainen MK, Lappalainen P. Actin-depolymerizing factor and cofilin-1 play overlapping roles in promoting rapid F-actin depolymerization in mammalian nonmuscle cells. Mol Biol Cell. 2005;16(2):649-64.
24. Po'uha ST, Shum MS, Goebel A, Bernard O, Kavallaris M. LIM-kinase 2, a regulator of actin dynamics, is involved in mitotic spindle integrity and sensitivity to microtubule-destabilizing drugs. Oncogene. 2010;29(4):597- 607.
25. Mardilovich K, Baugh M, Crighton D, Kowalczyk D, Gabrielsen M, Munro J, et al. LIM kinase inhibitors disrupt mitotic microtubule organization and impair tumor cell proliferation. Oncotarget. 2015;6(36):38469-86.
26. Contreras X, Mzoughi O, Gaston F, Peterlin MB, Bahraoui E. Protein kinase C-delta regulates HIV-1 replication at an early post-entry step in macrophages. Retrovirology. 2012;9:37.
27. Zhou S, Lin Q, Huang C, Luo X, Tian X, Liu C, et al. Rottlerin plays an antiviral role at early and late steps of Zika virus infection. Virol Sin. 2022;37(5):685-94.
28. Gobeil LA, Lodge R, Tremblay MJ. Macropinocytosis-like HIV-1 internalization in macrophages is CCR5 dependent and leads to efficient but delayed degradation in endosomal compartments. J Virol. 2013;87(2):735 -45.
29. Li M, Zhang D, Li C, Zheng Z, Fu M, Ni F, et al. Characterization of Zika Virus Endocytic Pathways in Human Glioblastoma Cells. Front Microbiol. 2020;11:242.
30. Daecke J, Fackler OT, Dittmar MT, Kräusslich HG. Involvement of clathrin-mediated endocytosis in human immunodeficiency virus type 1 entry. J Virol. 2005;79(3):1581-94.
31. Huang TY, DerMardirossian C, Bokoch GM. Cofilin phosphatases and regulation of actin dynamics. Curr Opin Cell Biol. 2006;18(1):26-31.
32. Bamburg JR. Proteins of the ADF/cofilin family: essential regulators of actin dynamics. Annu Rev Cell Dev Biol. 1999;15:185-230.
33. Prunier C, Prudent R, Kapur R, Sadoul K, Lafanechere L. LIM kinases: cofilin and beyond. Oncotarget. 2017;8(25):41749-63.
34. Yang N, Higuchi O, Ohashi K, Nagata K, Wada A, Kangawa K, et al. Cofilin phosphorylation by LIM- kinase 1 and its role in Rac-mediated actin reorganization. Nature. 1998;393(6687):809-12.
35. Gorovoy M, Niu J, Bernard O, Profirovic J, Minshall R, Neamu R, et al. LIM Kinase 1 Coordinates Microtubule Stability and Actin Polymerization in Human Endothelial Cells*. Journal of Biological Chemistry. 2005;280(28):26533-42.
36. Zhao H, Guo XK, Bi Y, Zhu Y, Feng WH. PKCdelta is required for porcine reproductive and respiratory syndrome virus replication. Virology. 2014;468-470:96-103.
37. Vorster PJ, Guo J, Yoder A, Wang W, Zheng Y, Xu X, et al. LIM kinase 1 modulates cortical actin and CXCR4 cycling and is activated by HIV-1 to initiate viral infection. J Biol Chem. 2011;286(14):12554-64.
38. Yi F, Guo J, Dabbagh D, Spear M, He S, Kehn-Hall K, et al. Discovery of Novel Small-Molecule Inhibitors of LIM Domain Kinase for Inhibiting HIV-1. J Virol. 2017;91(13).
39. Srivastava MC, Singh SW, Tewari JP. Anthelmintic activity of Mallotus philippinensis-kambila powder. Indian J Med Res. 1967;55(7):746-8.
40. Gschwendt M, Muller HJ, Kielbassa K, Zang R, Kittstein W, Rincke G, et al. Rottlerin, a novel protein kinase inhibitor. Biochem Biophys Res Commun. 1994;199(1):93-8.
41. Bain J, Plater L, Elliott M, Shpiro N, Hastie CJ, McLauchlan H, et al. The selectivity of protein kinase inhibitors: a further update. Biochem J. 2007;408(3):297-315.
42. Soltoff SP. Rottlerin Is a Mitochondrial Uncoupler That Decreases Cellular ATP Levels and Indirectly Blocks Protein Kinase C&#x3b4; Tyrosine Phosphorylation *. Journal of Biological Chemistry. 2001;276(41):37986 - 92.
43. Ojha D, Winkler CW, Leung JM, Woods TA, Chen CZ, Nair V, et al. Rottlerin inhibits La Crosse virus- induced encephalitis in mice and blocks release of replicating virus from the Golgi body in neurons. Nat Microbiol. 2021;6(11):1398-409.
44. Song KS, Kim JS, Yun EJ, Kim YR, Seo KS, Park JH, et al. Rottlerin induces autophagy and apoptotic cell death through a PKC-delta-independent pathway in HT1080 human fibrosarcoma cells: the protective role of autophagy in apoptosis. Autophagy. 2008;4(5):650-8.
45. Sugawara T, Nakatsu D, Kii H, Maiya N, Adachi A, Yamamoto A, et al. PKCdelta and epsilon regulate the morphological integrity of the ER-Golgi intermediate compartment (ERGIC) but not the anterograde and retrograde transports via the Golgi apparatus. Biochim Biophys Acta. 2012;1823(4):861-75.

**Formatted:** Font:

433

434 **Fig. 1. Rottlerin inhibits PKCδ-mediated PRRSV cellular entry.**
435 (A–C) MARC-145 cells were silenced with siRNA targeting PKCδ, or treated with rottlerin (5
436 μM), or EIPA (50 μM), inoculated with PRRSV FL12 (MOI = 1), and incubated for 48 hours at
437 37 °C in 5% CO2. (A and C) Virus titers in cell supernatants were quantified by qRT-PCR (A) or
438 titration (C). (B) Cells were washed with PBS, lysed, subjected to SDS-PAGE, and transferred
439 onto a nitrocellulose membrane. The blots were incubated with mouse anti-N monoclonal
440 antibodies or mouse anti-β-actin-HRP, followed by goat anti-mouse IgG-HRP. (D) Semi-confluent
441 MARC-145 cells were pretreated with DMSO, rottlerin (5 μM), or EIPA (50 μM) at 37°C for 2
442 hours, inoculated with PRRSV FL12 (MOI = 50), and incubated for 1 hour. Cells were washed
443 and treated with proteinase K to remove extracellular viruses for 45 minutes at 4°C. After fixation
444 and permeabilization, the cells were probed with anti-N monoclonal antibodies, followed by goat
445 anti-mouse IgG and Alexa Fluor 488 at 37 ℃. Nuclei were stained with Hoechst 33342.
446 Statistically significant differences were measured using Kruskal–Wallis nonparametric tests and
447 are indicated by asterisks (*, p < 0.05; **, p < 0.01). Scale bars, 5 μm. PKCδ, protein kinase Cδ;
448 PRRSV, porcine reproductive and respiratory syndrome virus; EIPA, 5-[*N*-ethyl-*N*-isopropyl]
449 amiloride; MOI, a multiplicity of infection; PBS, phosphate-buffered saline; SDS-PAGE, sodium
450 dodecyl sulfate-polyacrylamide gel electrophoresis; N, nucleocapsid; HRP, actin-horseradish
451 peroxidase; qRT-PCR, quantitative reverse transcription–polymerase chain reaction; DMSO,

452

453

dimethyl sulfoxide.

454 **Fig. 2. PRRSV enters MARC-145 cells via macropinocytosis and rottlerin blocks the**

**Formatted:** EndNote Bibliography, Line spacing: single

455 **macropinocytosis by decreasing actin polymerization.**
456 (A-C) Semi-confluent MARC-145 cells were pretreated with DMSO, rottlerin (5 μM), or EIPA
457 (50 μM) for 2 hours at 37 °C. (A) The cells were washed and then inoculated with CF®640R
458 dextran (10,000 MW) for 1 hour. Nuclei were stained with Hoechst 33342. (B-C) DMSO-, rottlerin
459 (5 μM)-, or EIPA (50 μM)-pretreated MARC-145 cells were washed and then inoculated with
460 PRRSV FL12 (MOI= 50) and CF®640R dextran (10,000 MW) for 1 hour. Cells were washed with
461 sodium acetate buffer (pH 5.2) to remove extracellular signals. After fixation and permeabilization,
462 cells were probed with anti-N monoclonal antibodies, followed by goat anti-mouse IgG and Alexa
463 Fluor 488 at 37 ℃. Nuclei were stained with Hoechst 33342. Scale bars, 5 μm. (C) Co-localization
464 of signals between dextran and PRRSV N protein in panel B was measured by Pearson’s
465 coefficient. (D) DMSO-, rottlerin (5 μM)-, or EIPA (50 μM)-pretreated MARC-145 cells were
466 inoculated with PRRSV FL12 (MOI= 50) and incubated with the indicated drug for 1 hour. After
467 fixation, cells were probed with CF®640R phalloidin at 37 ℃. Nuclei were stained with Hoechst
468 33342. Pseudopods resulting from actin rearrangement are marked as white arrows. (E) Cells with
469 clear pseudopods were counted as positive, whereas cells without pseudopods or with small rare
470 pseudopods were negative. Statistically significant differences are indicated by asterisks ( *, *p*-
471 value <0.05; **, *p*-value <0.01). DMSO, dimethyl sulfoxide; EIPA, 5-[*N*-ethyl-*N*-isopropyl]
472 amiloride; PRRSV, porcine reproductive and respiratory syndrome virus; MOI, multiplicity of

473

474

infection; N, nucleocapsid.

475 **Fig. 3. Rottlerin reverses PRRSV-induced activation of cofilin and LIMK1.**
476 (A and B) cofilin or LIMK1 was knocked down using siRNA targeting each protein. Then, cells
477 were inoculated with PRRSV FL12 (MOI = 1) for 1 hour and vigorously washed with PBS. After
478 2 days of incubation, cell lysates were subjected to SDS-PAGE, transferred onto nitrocellulose
479 membrane, and blotted for mouse anti-cofilin, rabbit anti-LIMK1, or mouse anti-β-actin-HRP,
480 followed by goat anti-rabbit IgG-HRP or goat anti-mouse IgG-HRP. (B) Viral levels in cell
481 supernatants of the cells were quantified by titration. (C-F) Confluent MARC-145 cells were
482 inoculated with PRRSV FL12 (MOI= 1) and incubated with rottlerin (5 μM) for the indicated time
483 period at 37 °C in 5% CO2. After washing with PBS, cell lysates were subjected to SDS-PAGE
484 and transferred onto a nitrocellulose membrane. The membranes were incubated with rabbit anti-
485 cofilin (P-cofilin, Ser3), mouse anti-cofilin (total cofilin), rabbit anti-LIMK1/2 (P-LIMK1,
486 Thr508/505), rabbit anti-LIMK1 (total LIMK1), or mouse anti-β-actin-HRP, followed by goat anti-
487 rabbit IgG-HRP or goat anti-mouse IgG-HRP. (D and F) Densitometry analysis plots of the kinetics
488 of the relative levels of P-LIMK1/total LIMK1 (D) or P-cofilin/total cofilin (F) after being
489 normalized by β-actin. PRRSV, porcine reproductive and respiratory syndrome virus; LIMK1,
490 LIM domain kinase 1; PBS, phosphate-buffered saline; SDS-PAGE, sodium dodecyl sulfate-
491 polyacrylamide gel electrophoresis; HRP, horseradish peroxidase; P-cofilin, phospho-cofilin; P-
492 LIMK1, phospho-LIMK1; siRNA, small interfering RNA; MOI, multiplicity of infection; mpi,

493

494

495

496

497

498

499

500

501

502

minute post-infection.

503

**Supporting information**

**Formatted:** Highlight

504

**S1. Uncropped western blot images.**

**Formatted:** Font: 12 pt, Highlight

**Formatted:** Font: 12 pt

**Formatted:** Font:

**Formatted:** 제목 11, Line spacing: single

Response to Reviewers

PONE-D-24-40479

Rottlerin inhibits macropinocytosis of Porcine Reproductive and Respiratory Syndrome Virus through the PKCδ-Cofilin signaling pathway

PLOS ONE

Dear Dr. Gianmarco Ferrara,

Thank you for giving us the opportunity to submit a revised draft of the manuscript “Rottlerin inhibits macropinocytosis of Porcine Reproductive and Respiratory Syndrome Virus through the PKCδ-Cofilin signaling pathway” for publication in PLOS ONE. We appreciate the time and effort that you dedicated to providing feedback on our manuscript based on journal requirements. We have incorporated all of your suggestions. Those changes are highlighted within the manuscript. Please see below, in red, for a point-by point response to the editor’s comments.

1. Please ensure that your manuscript meets PLOS ONE's style requirements, including those for file naming. The PLOS ONE style templates can be found at

[https://journals.plos.org/plosone/s/file?id=wjVg/PLOSOne_formatting_sample_main_](https://journals.plos.org/plosone/s/file?id=wjVg/PLOSOne_formatting_sample_main_body.pdf) [body.pdf](https://journals.plos.org/plosone/s/file?id=wjVg/PLOSOne_formatting_sample_main_body.pdf) and

[https://journals.plos.org/plosone/s/file?id=ba62/PLOSOne_formatting_sample_title_a](https://nam12.safelinks.protection.outlook.com/?url=https%3A%2F%2Fc05y1x9s.r.us-east-2.awstrack.me%2FL0%2Fhttps%3A%252F%252Fjournals.plos.org%252Fplosone%252Fs%252Ffile%253Fid%3Dba62%252FPLOSOne_formatting_sample_title_authors_affiliations.pdf%2F1%2F010f01922c9ed281-4e21a2fa-e487-4379-82ed-176097abd6e1-000000%2F_voNHBtkhz2RF_ZxJbii__nerL4%3D178&data=05%7C02%7Cchangin.oh%40yale.edu%7C09684ef41d5e4deb562008dcdde4f431%7Cdd8cbebb21394df8b4114e3e87abeb5c%7C0%7C0%7C638629224138494093%7CUnknown%7CTWFpbGZsb3d8eyJWIjoiMC4wLjAwMDAiLCJQIjoiV2luMzIiLCJBTiI6Ik1haWwiLCJXVCI6Mn0%3D%7C0%7C%7C%7C&sdata=U9Ahd2EF1SVJgxrTS6Hv%2FT0Z93PlkLWQrfezbbXgtE8%3D&reserved=0) [uthors_affiliations.pdf](https://nam12.safelinks.protection.outlook.com/?url=https%3A%2F%2Fc05y1x9s.r.us-east-2.awstrack.me%2FL0%2Fhttps%3A%252F%252Fjournals.plos.org%252Fplosone%252Fs%252Ffile%253Fid%3Dba62%252FPLOSOne_formatting_sample_title_authors_affiliations.pdf%2F1%2F010f01922c9ed281-4e21a2fa-e487-4379-82ed-176097abd6e1-000000%2F_voNHBtkhz2RF_ZxJbii__nerL4%3D178&data=05%7C02%7Cchangin.oh%40yale.edu%7C09684ef41d5e4deb562008dcdde4f431%7Cdd8cbebb21394df8b4114e3e87abeb5c%7C0%7C0%7C638629224138494093%7CUnknown%7CTWFpbGZsb3d8eyJWIjoiMC4wLjAwMDAiLCJQIjoiV2luMzIiLCJBTiI6Ik1haWwiLCJXVCI6Mn0%3D%7C0%7C%7C%7C&sdata=U9Ahd2EF1SVJgxrTS6Hv%2FT0Z93PlkLWQrfezbbXgtE8%3D&reserved=0).

Author response: Thank you for pointing this out. I corrected them and highlighted.

2. Thank you for stating in your Funding Statement:

“This research was supported by the Korea Institute of Planning and Evaluation for Technology in Food, Agriculture, and Forestry (IPET) through the Agriculture, Food, and Rural Affairs Convergence Technologies Program for Educating the Creative Global Leader Program, funded by the Ministry of Agriculture, Food, and Rural Affairs (MAFRA) (320005-04).”

Please provide an amended statement that declares *all* the funding or sources of support (whether external or internal to your organization) received during this study, as detailed online in our guide for authors

at <http://journals.plos.org/plosone/s/submit-now>. Please also include the statement “There was no additional external funding received for this study.” in your updated Funding Statement.

Please include your amended Funding Statement within your cover letter. We will change the online submission form on your behalf.

Author response: I revised the funding statement as follows:

“This research was supported by the Korea Institute of Planning and Evaluation for Technology in Food, Agriculture, and Forestry (IPET) through the Agriculture, Food,

and Rural Affairs Convergence Technologies Program for Educating the Creative Global Leader Program, funded by the Ministry of Agriculture, Food, and Rural Affairs (MAFRA) (320005-04).” There was no additional external funding received for this study.

3. Thank you for stating the following in the Acknowledgments Section of your manuscript:

“This research was supported by the Korea Institute of Planning and Evaluation for Technology in Food, Agriculture, and Forestry (IPET) through the Agriculture, Food, and Rural Affairs Convergence Technologies Program for Educating the Creative Global Leader Program, funded by the Ministry of Agriculture, Food, and Rural Affairs (MAFRA) (320005-04). We are deeply grateful to the KU Research Center for Zoonosis for providing advice on the design and progress of this study.”

We note that you have provided additional information within the Acknowledgements Section that is not currently declared in your Funding Statement. Please note that funding information should not appear in the Acknowledgments section or other areas of your manuscript. We will only publish funding information present in the Funding Statement section of the online submission form.

Please remove any funding-related text from the manuscript and let us know how you would like to update your Funding Statement. Currently, your Funding Statement reads as follows:

“This research was supported by the Korea Institute of Planning and Evaluation for Technology in Food, Agriculture, and Forestry (IPET) through the Agriculture, Food, and Rural Affairs Convergence Technologies Program for Educating the Creative Global Leader Program, funded by the Ministry of Agriculture, Food, and Rural Affairs (MAFRA) (320005-04).”

Please include your amended statements within your cover letter; we will change the online submission form on your behalf.

Author response: Thank you for pointing this out. I revised the Acknowledgements as follows. I revised the manuscript as well.:

We are deeply grateful to the KU Research Center for Zoonosis for providing advice on the design and progress of this study.

4. We note that your Data Availability Statement is currently as follows: [All relevant data are within the manuscript and its Supporting Information files.]

Please confirm at this time whether or not your submission contains all raw data required to replicate the results of your study. Authors must share the “minimal data

set” for their submission. PLOS defines the minimal data set to consist of the data required to replicate all study findings reported in the article, as well as related metadata and methods (https://journals.plos.org/plosone/s/data-availability#loc- minimal-data-set-definition).

For example, authors should submit the following data:

- The values behind the means, standard deviations and other measures reported;
- The values used to build graphs;
- The points extracted from images for analysis.

Authors do not need to submit their entire data set if only a portion of the data was used in the reported study.

If your submission does not contain these data, please either upload them as Supporting Information files or deposit them to a stable, public repository and provide us with the relevant URLs, DOIs, or accession numbers. For a list of recommended repositories, please see [https://journals.plos.org/plosone/s/recommended-](https://journals.plos.org/plosone/s/recommended-repositories) [repositories](https://journals.plos.org/plosone/s/recommended-repositories).

If there are ethical or legal restrictions on sharing a de-identified data set, please explain them in detail (e.g., data contain potentially sensitive information, data are owned by a third-party organization, etc.) and who has imposed them (e.g., an ethics committee). Please also provide contact information for a data access committee, ethics committee, or other institutional body to which data requests may be sent. If data are owned by a third party, please indicate how others may request data access.

Author response: Thank you for pointing this out. I would like to revise my Data Availability Statement as follows:

“The minimal data set is included within the manuscript and its supporting information files.”

5. PLOS ONE now requires that authors provide the original uncropped and unadjusted images underlying all blot or gel results reported in a submission’s figures or Supporting Information files. This policy and the journal’s other requirements for blot/gel reporting and figure preparation are described in detail at [https://journals.plos.org/plosone/s/figures#loc-blot-and-gel-reporting-](https://journals.plos.org/plosone/s/figures#loc-blot-and-gel-reporting-requirements)

[requirements](https://journals.plos.org/plosone/s/figures#loc-blot-and-gel-reporting-requirements) and [https://journals.plos.org/plosone/s/figures#loc-preparing-figures-](https://journals.plos.org/plosone/s/figures#loc-preparing-figures-from-image-files) [from-image-files](https://journals.plos.org/plosone/s/figures#loc-preparing-figures-from-image-files). When you submit your revised manuscript, please ensure that your figures adhere fully to these guidelines and provide the original underlying images for all blot or gel data reported in your submission. See the following link for instructions on providing the original image data: [https://journals.plos.org/plosone/s/figures#loc-](https://journals.plos.org/plosone/s/figures#loc-original-images-for-blots-and-gels) [original-images-for-blots-and-gels](https://journals.plos.org/plosone/s/figures#loc-original-images-for-blots-and-gels).

In your cover letter, please note whether your blot/gel image data are in Supporting Information or posted at a public data repository, provide the repository URL if

relevant, and provide specific details as to which raw blot/gel images, if any, are not available. Email us at [plosone@plos.org](mailto:plosone@plos.org) if you have any questions.

Author response: I have included my original uncropped and unadjusted images underlying all blot or gel results in the Supporting information file.

Additional Editor Comments (if provided):

Dear authors, before the article can go into revision, it is necessary that you upload the full blots (including molecular weights and merges) of all the gels that make up the figures. Furthermore, excessive contrast of the bands in these figures is not recommended. Please review the manuscript and resubmit.

[Note: HTML markup is below. Please do not edit.]

[NOTE: If reviewer comments were submitted as an attachment file, they will be attached to this email and accessible via the submission site. Please log into your account, locate the manuscript record, and check for the action link "View Attachments". If this link does not appear, there are no attachment files.]

While revising your submission, please upload your figure files to the Preflight Analysis and Conversion Engine (PACE) digital diagnostic

tool, [https://pacev2.apexcovantage.com/](https://nam12.safelinks.protection.outlook.com/?url=https%3A%2F%2Fc05y1x9s.r.us-east-2.awstrack.me%2FL0%2Fhttps%3A%252F%252Fpacev2.apexcovantage.com%252F%2F1%2F010f01922c9ed281-4e21a2fa-e487-4379-82ed-176097abd6e1-000000%2Frpon0Y0KWCrdkW8H4cCFwAcatMQ%3D178&data=05%7C02%7Cchangin.oh%40yale.edu%7C09684ef41d5e4deb562008dcdde4f431%7Cdd8cbebb21394df8b4114e3e87abeb5c%7C0%7C0%7C638629224138510052%7CUnknown%7CTWFpbGZsb3d8eyJWIjoiMC4wLjAwMDAiLCJQIjoiV2luMzIiLCJBTiI6Ik1haWwiLCJXVCI6Mn0%3D%7C0%7C%7C%7C&sdata=%2B0ZDlzM7JppbTUi46njQo3ffIOiMfACvA%2Fgv5xaTrUM%3D&reserved=0). PACE helps ensure that figures meet PLOS requirements. To use PACE, you must first register as a user. Registration is free. Then, login and navigate to the UPLOAD tab, where you will find detailed instructions on how to use the tool. If you encounter any issues or have any questions when using PACE, please email PLOS at [figures@plos.org](mailto:figures@plos.org). Please note that Supporting Information files do not need this step.

Thank you for your consideration of our submission. We look forward to the possibility of sharing our research with your readers.

Sincerely,

Changin Oh, DVM, PhD Associate research scientist Department of Genetics

Yale University School of Medicine

New Haven Connecticut 06520-8005, USA e-mail: [changin.oh@yale.edu](mailto:changin.oh@yale.edu)
